# Neuroinflammation, autoinflammation, splenomegaly and anemia caused by bi-allelic mutations in *IRAK4*

**DOI:** 10.3389/fimmu.2023.1231749

**Published:** 2023-09-06

**Authors:** Samantha Cooray, Fiona Price-Kuehne, Ying Hong, Ebun Omoyinmi, Alice Burleigh, Kimberly C. Gilmour, Bilal Ahmad, Sangdun Choi, Mohammad W. Bahar, Paul Torpiano, Andrey Gagunashvili, Barbara Jensen, Evangelos Bellos, Vanessa Sancho-Shimizu, Jethro A. Herberg, Kshitij Mankad, Atul Kumar, Marios Kaliakatsos, Austen J. J. Worth, Despina Eleftheriou, Elizabeth Whittaker, Paul A. Brogan

**Affiliations:** ^1^ Infection, Immunity and Inflammation Department, University College London Great Ormond Street Institute of Child Health, London, United Kingdom; ^2^ Centre for Adolescent Rheumatology Versus Arthritis, University College London, London, United Kingdom; ^3^ Department of Immunology and Gene Therapy, Great Ormond Street Hospital for Children NHS Foundation Trust, London, United Kingdom; ^4^ Department of Molecular Science and Technology, Ajou University, Suwon, Republic of Korea; ^5^ Division of Structural Biology, University of Oxford, The Wellcome Centre for Human Genetics, Oxford, United Kingdom; ^6^ Faculty of Life and Environmental Sciences, University of Iceland, Reykjavík, Iceland; ^7^ Section of Paediatric Infectious Diseases, Imperial College London, London, United Kingdom; ^8^ Centre for Paediatrics and Child Health, Faculty of Medicine, Imperial College London, London, United Kingdom; ^9^ Department of Paediatric Infectious Diseases, St Mary’s Hospital, Imperial College NHS Healthcare Trust, London, United Kingdom; ^10^ Department of Radiology, Great Ormond Street Hospital for Children NHS Foundation Trust, London, United Kingdom; ^11^ Department of Histopathology, Great Ormond Street Hospital for Children NHS Foundation Trust, London, United Kingdom; ^12^ Department of Neurology, Great Ormond Street Hospital for Children NHS Foundation Trust, London, United Kingdom

**Keywords:** autoinflammation, neuroinflammation, IRAK-4, toll-like receptor, splenomegaly, anemia, NASA

## Abstract

We describe a novel, severe autoinflammatory syndrome characterized by neuroinflammation, systemic autoinflammation, splenomegaly, and anemia (NASA) caused by bi-allelic mutations in *IRAK4*. IRAK-4 is a serine/threonine kinase with a pivotal role in innate immune signaling from toll-like receptors and production of pro-inflammatory cytokines. In humans, bi-allelic mutations in *IRAK4* result in IRAK-4 deficiency and increased susceptibility to pyogenic bacterial infections, but autoinflammation has never been described. We describe 5 affected patients from 2 unrelated families with compound heterozygous mutations in *IRAK4* (c.C877T (p.Q293*)/c.G958T (p.D320Y); and c.A86C (p.Q29P)/c.161 + 1G>A) resulting in severe systemic autoinflammation, massive splenomegaly and severe transfusion dependent anemia and, in 3/5 cases, severe neuroinflammation and seizures. IRAK-4 protein expression was reduced in peripheral blood mononuclear cells (PBMC) in affected patients. Immunological analysis demonstrated elevated serum tumor necrosis factor (TNF), interleukin (IL) 1 beta (IL-1β), IL-6, IL-8, interferon α2a (IFN-α2a), and interferon β (IFN-β); and elevated cerebrospinal fluid (CSF) IL-6 without elevation of CSF IFN-α despite perturbed interferon gene signature. Mutations were located within the death domain (DD; p.Q29P and splice site mutation c.161 + 1G>A) and kinase domain (p.Q293*/p.D320Y) of IRAK-4. Structure-based modeling of the DD mutation p.Q29P showed alteration in the alignment of a loop within the DD with loss of contact distance and hydrogen bond interactions with IRAK-1/2 within the myddosome complex. The kinase domain mutation p.D320Y was predicted to stabilize interactions within the kinase active site. While precise mechanisms of autoinflammation in NASA remain uncertain, we speculate that loss of negative regulation of IRAK-4 and IRAK-1; dysregulation of myddosome assembly and disassembly; or kinase active site instability may drive dysregulated IL-6 and TNF production. Blockade of IL-6 resulted in immediate and complete amelioration of systemic autoinflammation and anemia in all 5 patients treated; however, neuroinflammation has, so far proven recalcitrant to IL-6 blockade and the janus kinase (JAK) inhibitor baricitinib, likely due to lack of central nervous system penetration of both drugs. We therefore highlight that bi-allelic mutation in *IRAK4* may be associated with a severe and complex autoinflammatory and neuroinflammatory phenotype that we have called NASA (neuroinflammation, autoinflammation, splenomegaly and anemia), in addition to immunodeficiency in humans.

## Introduction

Monogenic autoinflammatory diseases are a group of rare disorders characterized by recurrent fevers and caused by mutations in genes involved in innate immune signaling (1). These include (among many others) TNF receptor associated periodic syndrome (TRAPS), deficiency of IL-1 receptor antagonist (DIRA), cryopyrin-associated periodic syndromes (CAPS), as well as type-1 interferonopathies, where there is specific dysregulation of interferon-related signaling. Genetic evaluation continues to reveal causative mutations in an ever-expanding list of innate immune-signaling molecules.

Toll like receptors (TLRs) and interleukin-1 (IL-1) receptors (IL-1Rs) are a large family of transmembrane receptors that play an important role in innate immune signaling and inflammation (2). TLRs are pattern recognition receptors (PRRs) that recognize pathogen-associated molecular patterns (PAMPS). IL-1Rs are activated by cognate cytokines, including IL-1β and IL-18, essential in innate immune responses (3). Interleukin-1 (IL-1) receptor-associated kinase-4 (IRAK-4) is a serine/threonine kinase and member of a family of kinases that play a pivotal role in TLR and IL-1R mediated innate immune signaling and inflammation (4). Upon activation of TLRs by PAMPs such as lipopolysaccharide (LPS), flagellin and unmethylated CpG DNA, the receptors oligomerize at the plasma or endosomal membrane, allowing association of their C-terminal Toll interleukin-1 receptor (TIR) domains (2). This results in recruitment of adaptor protein MyD88 and IRAK-4 via their death domains (DD) into a myddosome complex resulting in the auto- and *trans*-phosphorylation of IRAK-4 and subsequent recruitment and activation of IRAK-1/2 (5, 6). Downstream signaling via the IκB kinase (IKK) complex and mitogen-activated protein kinases (MAPKs) results in activation of NF-κB and of AP-1 transcription factors, respectively and an increase in pro-inflammatory cytokines (1). Downstream of TLRs 7 and 9, IRAK-4 activation also leads to the activation of IRF7 by IKKα and/or IRAK1 leading to upregulation of type I interferons (7).

Bi-allelic recessive mutations in *IRAK4* in humans described to date are loss-of-function and result in a clinical phenotype of immunodeficiency with variable susceptibility to infection with pyogenic bacteria (8–10). These mutations result in loss of protein expression and a lack of response to TLR stimuli with abrogation of downstream protective pro-inflammatory signals. MyD88 deficiency has also been described which results in an indistinguishable clinical phenotype (10). Autoinflammation has never been described in association with mutations in *IRAK4*, although upregulation of IRAK-4 has been implicated in inflammation in murine models of lupus and rheumatoid arthritis, which has led to the development of novel selective IRAK-4 inhibitors (11, 12). Missense mutations have been demonstrated in TLR8 leading to upregulation of NF-κB and interferon signaling resulting in a hyperinflammatory phenotype with bone marrow failure or severe autoimmune hemolytic anemia with central nervous system (CNS) vasculitis (13, 14). However, missense mutation in *IRAK4* leading to autoinflammation has never been described.

Here we describe 5 patients from 2 unrelated kindreds with bi-allelic mutations in *IRAK4*, where one of which was a missense mutation, resulting in a severe autoinflammatory phenotype without overt immune deficiency, presenting with fever without infection, increased inflammatory markers, massive splenomegaly, transfusion dependent anemia; and severe neuroinflammation in 3/5 cases.

## Methods

### Study participants

All participants were referred to Great Ormond Street Hospital NHS Foundation Trust (GOSH), a quaternary referral center for the investigation and treatment of pediatric autoinflammatory disease. All work involving human subject research was approved by the GOSH Research Ethics Committee (REC reference 08/H0713/82). All adult subjects provided written informed consent to participate; parental consent was obtained for all children involved in the study; and assent obtained where appropriate. Control sera were obtained from adolescent healthy control subjects with local ethics approval (REC reference II/LP/0330).

### Whole exome sequencing and genetic analysis

Whole exome sequencing (WES) was carried out for all study subjects with Sanger sequencing confirmation. The output variant call format (VCF) file was annotated using wANNOVAR, the web-based ANNOVAR tool from Wang Genomic Labs (15). Potential disease-causing variants in the WES data were explored using Exomiser (16) using the following human phenotype ontology terms (HPO): hepatosplenomegaly (HP:0001433), short stature (HP:0004322), elevated circulating C-reactive protein (CRP) concentration (HP:0011227), elevated erythrocyte sedimentation rate (ESR) (HP:0003565) and anemia (HP:0001903). MutationTaster and NNsplice were used to analyze the impact of the c.161 + 1 G>A splice-site mutation (17).

Sanger sequencing of *IRAK4* exon 2 (Family B) and exons 8 and 9 (Family A) were carried out using forward and reverse primers:

Exon 2 - TCTTTATTATGGTATAATCAGTTGCTG and TCCTCCATAGTGGAGAGGTTTAC;

Exon 8 - AACATCATCTTCAGTTGTTGCC and AGACCTGCATATGAATCGTGAATAG;

Exon 9 - TGATGCATACTATAAAACGTTACACTC and ACATAAGAGAGATAAACAAGATGGG.

Sanger chromatograms were produced using CodonCode Aligner V10.0.02 and sequencing confirmed the segregation of *IRAK4* variants within both families.

### L-selectin shedding assay

CD62L (L-selectin) shedding assays were carried out to test the function of IRAK-4, as previously described (18). Briefly, 100 μl of heparinized whole blood was either unstimulated in phosphate buffered saline (PBS) or stimulated with TLR agonists: TLR4 – LPS 120 ng/mL (Sigma-Aldrich, L-9764); TLR1/2 – Pam3CSK4 100 ng/mL, TLR2/6 – MALP-2 100 ng/mL, TLR7/8 – R-848 resiquimod hydrochloride 3 μg/mL, TLR5 – flagellin 1 μg/mL (all from Enzo Life Sciences, ALX-165-066-M002, ALX-162-027-C050, ALX-165-066-M002, ALX-522-058-C010, respectively), or phorbolmyristyl acetate (PMA) 20 ng/mL (Sigma, P-8139) for 30 minutes at 37°C. Cells were then incubated with fluorescein isothiocyanate–conjugated (FITC) mouse anti-human CD62L monoclonal antibody 10 μL and phycoerythrin (PE) mouse anti-human CD11b monoclonal antibody 10 μL (BD Biosciences Pharmingen, 555543, 340712) for 30 mins on ice. Erythrocytes were lysed with FACS lysis buffer (BD Biosciences Pharmingen, 349242). CD62L expression was quantified in granulocytes by flow cytometry using a FACS Caliber flow cytometer and analyzed using BD FACSuite™ (BD Life Sciences).

### Interferon-stimulated gene signature (ISG) assay

The ISG assay was carried out as previously described (19). CXCL10, CXCL9, IFI27, IFI44L, IFIT1, IFNB1, IFNγ, IL-18, RSAD2, SIGLEC1, ISG15 genes were amplified using 2 μl QuantiTect Primer Assay primers (Qiagen, QT00099274, QT00072814, QT00051457, QT00201012, QT00005271, QT00008134, QT00203763, QT00000525, QT1003065, QT00013461, QT00014560) 10 μl iTaq Universal SYBR Green Supermix (BioRad, 1725121), 6 μl nuclease-free water (Invitrogen, AM9937) and 2 μl cDNA using a real-time quantitative PCR CFX96 Touch thermal cycler (Bio-Rad) and cycle threshold (Ct) values analyzed using CFX Manager™ 3.1 Software (Bio-Rad, 1845000).

### Multiple sequence alignment

IRAK-4 amino acid sequences for *Homo sapiens* (NP_001107654.1), *Pan troglodytes* (XP_001166075.1), *Macaca mulatta* (NP_001129573.1), *Rattus norvegicus* (NP_001100261.2) *Equus caballus* (XP_001488489.1) and *Taeniopygia guttata* (XP_002194205.1) were retrieved from the National Center for Biotechnology Information (NCBI) and aligned using Clustal Omega at EMBL-EBI (20).

### Structural modeling of IRAK-4 mutations

The amino acid sequence of human IRAK-4 was obtained from the NCBI (accession no. AAM15772.1). Three-dimensional (3D) structural modeling of p.Q29P and p.D320Y mutations within the DD and kinase domain (KD) of human IRAK-4, respectively, was performed using AlphaFold v.2 on Google CoLab (21, 22). The p.Q29P mutant (residues 1-94) was compared to a model of the human wild-type IRAK-4 DD using the RSCB protein data bank (PDB) structural alignment tool. Comparison of the interactions of the DD of wild-type and p.Q29P IRAK-4 proteins with the DD of IRAK-2 (residues 1-80) [PDB: 3MOP] were performed by superposition of p.Q29P and wild-type IRAK-4 using the structure alignment tools within Coot (23). Schematic representation of DD domains of MyD88, IRAK-2 and wild-type and p.Q29P versions of IRAK-4 were based on the helical structure and assembly model reported by Lin et al. (5). The model of p.D320Y KD mutant was compared to the published crystal structure of the human IRAK-4 KD (PDB code 2NRU) (24).

### Protein contacts analysis

The p.D320Y mutant of IRAK-4 was constructed using PyRosetta (25) as previously described (26). In order to investigate the extent of intra-hydrogen bonds, metals, ionic, arene, and covalent bonds, as well as van der Waals distance interactions, the contact patterns of IRAK-4 wild-type and mutant p.D320Y proteins were analyzed. MOE2020 was used to analyze the intra-residue contacts (27).

### Peripheral blood mononuclear cell isolation

Peripheral blood mononuclear cells (PBMC) were isolated from freshly-drawn heparinized whole blood from patients and controls by gradient density centrifugation using Lymphoprep™ (STEMCELL™ Technology), according to the manufacturer’s instructions.

### IRAK-4 protein expression analysis

Unstimulated PBMC were fixed and permeabilized with BD Cytofix/Cytoperm™ (BD Biosciences, 554713) and stained with PE mouse anti-human IRAK-4 monoclonal antibody (BD Biosciences, 560303). IRAK-4 expression was quantified by flow cytometry using a CytoFLEX flow cytometer (Beckman Coulter) and analyzed using FlowJo™ software (BD Life Sciences).

### Measurement of cytokines

TNF, IL-1β, IL-6, IL-8, IFN-α2a, IFN-β and IFN-γ cytokine levels were measured in sera of all members of both families and healthy controls using an electrochemiluminescence immunoassay Meso Scale Discovery (MSD) multiplex kit (Meso Scale Diagnostics), as per manufacturer’s instructions. CSF cytokines were assayed by the routine hospital laboratory using the Cytometric Bead Array (BD Biosciences) with the exception of interferon-alpha which was outsourced to a reference laboratory in France (Hopitaux Universitaires Paris Centre).

PBMC were stimulated with 100 ng/ml LPS (Sigma-Aldrich, L2630) for 6 hours and IL-6 and TNF in the supernatants were quantified using enzyme-linked immunoassay (ELISA) for IL-6 (BioLegend, 430515) and TNF (Thermo Fisher, 88-7346-88) as per manufacturer’s instructions.

### NF-κB p65, STAT1 and STAT3 phosphorylation assays

PBMC were incubated with live/dead fixable violet dead cell stain kit (Invitrogen, L34955) for 30 minutes. Cells were then treated with either 100 ng/ml TNF (RayBiotech, 230-00243-50), LPS (Sigma Aldrich, L2880) or IFN-α2b (GenScript, 203002-50), or 1 µg/ml IL-1β (Aviscera Bioscience, 00756-01-59), for up to 60 minutes. Cells were fixed with BD Phosflow Fix Buffer (BD Biosciences, 557870), permeabilized with BD Perm Buffer III (Biosciences, 558050) and stained with the following antibodies: PE anti-phospho-STAT1 (pY701) (BD Biosciences, 612564); Alexa Fluor^®^ 488-anti-phospho-STAT3 (pY705) (Becton Dickinson 557814); or PE anti-NF-κB phospho-p65 (pS529) (BD Biosciences, 558423) and quantified by flow cytometry as described above.

### Statistical analysis

Summative numeric data were expressed as mean (standard error of mean) unless otherwise specified. Comparative statistical analyses (ANOVA/T-test) were performed using GraphPad Prism Software version 9.5.0.

## Results

### Clinical presentation

**Figure 1:**
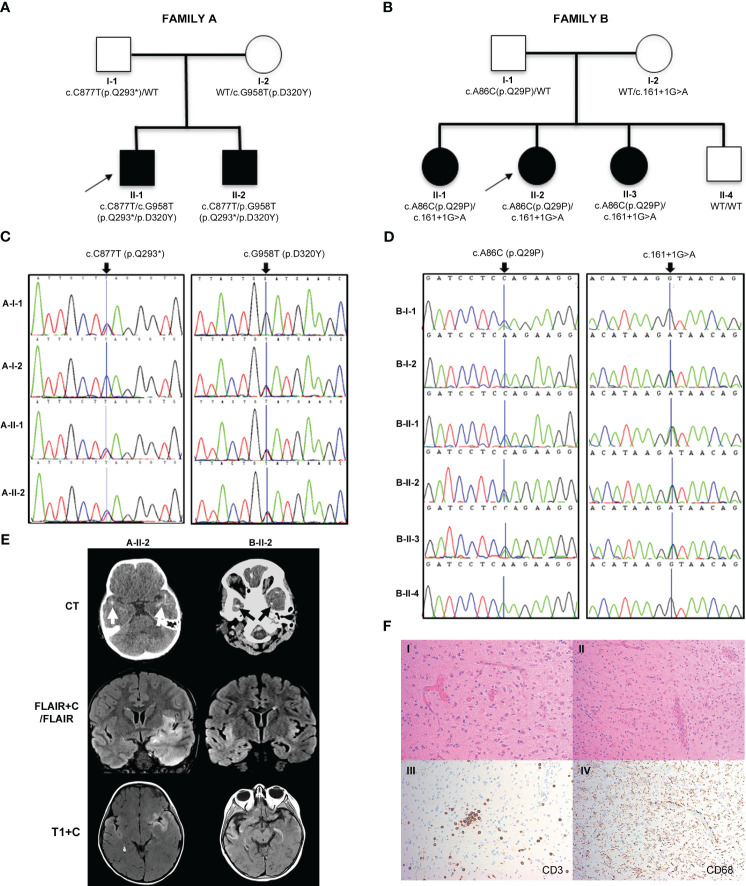
Pedigrees, genetic analysis, neurological features and shedding assay results in families A and B. **(A, B)** Pedigrees of family A and family B, respectively, with black shapes showing affected children, white shapes showing unaffected parents and children and arrows indicating the proband of each family. Genetic testing revealed that the affected children in family A were compound heterozygous for *IRAK4* gene mutations c.C877T (p.Q293*) and c.G958T (p.D320Y) and in family B were compound heterozygous for *IRAK4* gene mutations c.A86C (p.Q29P) and c.161 + 1G>A. Parents in both families were found to be heterozygotes. **(C)** Sanger sequencing electropherograms for family A showing the c.C877T (p.Q293*) variant inherited from the father (A-I-1), and c.G958T (p.D320Y) variant inherited from the mother (A-I-2). **(D)** Sanger sequencing electropherogram for family B showing c.A86C (p.Q29P) variant inherited from the father (B-I-1), and c.161 + 1G>A variant inherited from the mother (B-I-2). **(E)** Brain imaging. Left column (A-II-2): Axial CT scan showing bitemporal subcortical calcification (arrows). Post-contrast coronal FLAIR (FLAIR+C) and axial T1-weighted (T1+C) MRI sequences, showing asymmetrical swelling, signal abnormality and cortical-subcortical contrast enhancement in temporal lobes, insular and basal frontal regions. Right column (B-II-2): CT scan showing patchy subcortical calcification in the temporal lobes bilaterally (arrows). Coronal MRI FLAIR demonstrates asymmetrical signal abnormality involving the insular regions and temporal lobes. Post-contrast T1-weighted (T1+C) MRI performed after 3 months shows persisting cortical and subcortical contrast enhancement with signal abnormality in the temporal lobes and the frontal basal regions. **(F)** Brain biopsy of B-II-2 showing disruption of the cortical architecture with interspersed reactive astrocytes in keeping with gliosis (I); blood vessels with reactive endothelium and scattered mononuclear chronic inflammation (II); scattered CD3 positive T lymphocytes (III); diffuse microglial upregulation with interspersed macrophages (IV).

In family A (**Figure 1A**), two boys (A-II-1) and (A-II-2) born to non-consanguineous parents (Caucasian father and Chinese mother) presented aged 23 months and 24 months. Both children had been previously well with normal growth and development, other than a few short episodes of self-resolving pyrexia without obvious infectious focus.

The proband (A-II-1) presented with recurrent episodes of fever >39°C without infectious focus, massive splenomegaly, elevated inflammatory markers and severe hypochromic microcytic anemia (**Table 1**, **Figure 1A**; **Supplementary Table 1**). Clinical examination revealed hepatomegaly with massive splenomegaly in the absence of generalized lymphadenopathy (**Table 1**). Chest x-ray and head magnetic resonance imaging (MRI) were normal (**Supplementary Table 2**). He was treated on multiple occasions with intravenous (IV) antibiotics for presumed infection and packed red blood cell (RBC) transfusions for presumed hemolysis. However, extensive microbiological screens for viral, bacteria, fungal and parasitic infections were consistently negative, with the exception of one *staphylococcus aureus* from a single sputum culture. Bone marrow aspirate showed increased erythroid activity but no evidence of malignancy or hemophagocytosis (**Supplementary Tables 1**, **3**).

**Table 1:** Patient genotype, phenotype, demographics and laboratory results.

	FAMILY A					FAMILY B					
	I-1		I-2	II-1	II-2	I-1	I-2	II-1	II-2	II-3	II-4
Genotype *IRAK4* (HGVS cDNA)	WT/c.C877T		WT/c.G958T	c.G958T/c.C877T	c.G958T/c.C877T	WT/c.A86C	WT/ c.161 + 1G>A	c.A86C/c.161 + 1G>A	c.A86C/c.161 + 1G>A	c.A86C/c.161 + 1G>A	WT/WT
IRAK-4 (HGVS protein)	WT/p.Q293*		WT/p.D320Y	p.Q293*/p.D320Y	p.Q293*/p.D320Y	WT/p.Q29P	WT/-	p.Q29P/-	p.Q29P/-	p.Q29P/-	WT
Functional impact of variant	Deleterious: premature stop codon		Unknown	Deleterious: premature stop codon/Unknown	Deleterious: premature stop codon/Unknown	Unknown	Deleterious: abolition of splice site	Unknown/ Deleterious: abolition of splice site	Unknown/ Deleterious abolition of splice site	Unknown/ Deleterious abolition of splice site	N/A
Zygosity	Heterozygous		Heterozygous	Compound heterozygous	Compound heterozygous	Heterozygous	Compound heterozygous	Compound heterozygous	Compound heterozygous	Compound heterozygous	WT
Affected	No		No	Yes	Yes	No	No	Yes	Yes	Yes	No
Demographics											
Ethnicity	White British		Chinese	Mixed	Mixed	Syrian	White Polish	Mixed	Mixed	Mixed	Mixed
Sex	M		F	M	M	M	F	F	F	F	M
Age at first presentation	N/A		N/A	23 months	24 months	N/A	N/A	8 weeks	3 weeks	Neonate	N/A
Clinical Features											
Recurrent fevers	N/A		N/A	Yes	Yes	N/A	N/A	Yes	Yes	Yes	No
Persistently elevated inflammatory markers	N/A		N/A	Yes	Yes	N/A	N/A	Yes	Yes	Yes	No
Hepatomegaly	N/A		N/A	Yes	Yes	N/A	N/A	Yes	Yes	No	No
Splenomegaly	N/A		N/A	Yes	Yes	N/A	N/A	Yes	Yes	Yes	No
Anemia	N/A		N/A	Yes	Yes	N/A	N/A	Yes	Yes	Yes	No
Transaminitis	N/A		N/A	Yes	Yes	N/A	N/A	Yes	Yes	UK	No
Biochemical features								pre/post- splenectomy			
Hb (g/L; RR 115-145)	NI		NI	76	95	NI	NI	43/109	64	80	129
CRP (mg/L; RR 0-20)	NI		NI	125	49	NI	NI	107/85	60	39	<5
ESR (mm/hr; RR 0-10)	NI		NI	135	116	NI	NI	UK/127	343	100	6
SAA (mg/L; RR <10)	NI		NI	101	196	NI	NI	UK/97	58	202	3.5
ALT (U/L; RR 10-25)	NI		NI	68	46	NI	NI	<6/58	86	43	32
Imaging											
Abdominal ultrasound	NI		NI	Hepato- splenomegaly	Hepato- splenomegaly	NI	NI	Hepato- splenomegaly	Hepato- splenomegaly	Splenomegaly	NI
CT - head	NI		NI	NI	Bilateral temporal subcortical calcifications.	NI	NI	NI	Bilateral temporal cortical calcifications.	NI	NI
MRI head	NI		NI	Past choroid plexus hemorrhage. No calcification.	Bilateral (left>right) temporal encephalitis with vasogenic oedema and subcortical and cortical enhancement.	NI	NI	Normal	Bilateral temporal cortical hyperintensities & enhancement. Volume reduction of right temporal lobe and hippocampus.	Symmetrical signal abnormality in the medial temporal regions and white matter adjacent to frontal horns	NI

RR, Reference Range; UK, Unknown; N/A, Not applicable; NI, Not indicated; ND, Not done; Hb, hemoglobin; RBC, red blood cells; CRP, C-reactive protein; ESR, erythrocyte sedimentation rate; SAA, serum amyloid A; ALT, alanine aminotransaminase; CT, computed tomography; MRI, magnetic resonance imaging.

Immunological work up revealed persistently elevated inflammatory markers and anemia, elevated IgG and IgM but normal lymphocyte subsets and vaccine responses, and negative autoantibody screens (**Supplementary Tables 1**, **3**). He was suspected of having an undiagnosed autoinflammatory condition and commenced on empiric therapeutic trials of anti-IL-1 agents anakinra followed by canakinumab, which had no significant clinical impact, or impact on laboratory indices of acute phase response (**Supplementary Figure 1A** and **Supplementary Table 2**). He was switched to the anti-IL-6R monoclonal antibody tocilizumab aged 5 years (162 mg subcutaneously [8.1 mg/kg] every 3 weeks based on a modified Still’s disease dosing protocol for children) which resulted in an immediate, complete, and sustained clinical and serological resolution of systemic inflammation (cessation of fevers and complete normalization of inflammatory markers), and complete resolution of anemia (**Supplementary Figure 1A** and **Supplementary Table 2** **)**. He has remained well at 4 years follow up on the same very modest tocilizumab dosing regimen, with normal growth, and normal inflammatory markers; splenomegaly has persisted, however. There have been no serious infectious episodes over this time-period, including complete recovery from a mild SARS-CoV-2 infection. Magnetic resonance imaging (MRI) of the brain, to screen for neuroinflammation in the absence of symptoms, was normal.

The brother of the proband, A-II-2 presented with fever, hepatosplenomegaly, anemia and transaminitis following a short coryzal illness. In common with his brother, immunological tests were normal apart from an elevated IgG (**Supplementary Table 3**). He was commenced on tocilizumab (162 mg [12 mg/kg] s-c every 3 weeks) and initially had a complete clinical and serological acute phase response with improved growth after 4 months (**Supplementary Table 2** and **Supplementary Figure 1B**). He remained completely well until 5 years of age (approximately 3 years after starting tocilizumab), when he re-presented with seizures. CT brain imaging revealed bilateral temporal subcortical calcifications and MRI brain showed vasogenic oedema with subcortical and cortical enhancement (**Figure 1E**). Electroencephalogram (EEG) showed slow and high amplitude cortical rhythms in keeping with a mild-moderate encephalopathy of non-specific etiology. Extensive microbiological screens were carried out in blood and CSF, including culture and metagenomic analysis for viral, bacterial and fungal pathogens, which were all negative (**Supplementary Tables 3** and **4**). The cerebrospinal fluid (CSF) was also negative for encephalitis-associated autoantibodies and paraneoplastic antibodies and had normal levels of neurotransmitters (**Supplementary Table 4**). High IL-6 levels were found in the CSF (225 pg/ml; RR<50) and blood and ISG assay demonstrated elevated IFI27 (copy number threshold 6.04; RR 0.09 – 2.24) which was also found in his older brother (**Supplementary Table 3**). He was commenced on levetiracetam and tocilizumab dosing was increased to 2-weekly from 3-weekly (**Supplementary Table 2**). However, worsening neuroinflammation was clearly demonstrated on brain MRI with ongoing seizures so he was switched to baricitinib 4 mg bd (monogenic interferonopathy dosing regimen, **Supplementary Table 2**). However, after 3 months CNS inflammation persisted (ongoing seizures with evidence of persistent neuroinflammation on repeat brain MRI), anemia returned, and acute phase reactants deteriorated (ESR 60 mm/h; SAA 82.9 mg/L; CRP 9 mg/L; **Supplementary Figure 1B**). He was unable to be weaned off prednisolone (0.25 mg/kg/day) and had developed significant glucocorticoid toxicity (weight gain and behavioral disturbance). Thus, at the time of writing, he is currently being converted back to tocilizumab (162 mg every 2 weeks) in combination with an alternative immunosuppressive agent (mycophenolate mofetil 600 mg/m^2^ twice daily) and is currently being worked up for consideration of allogeneic hematopoietic stem cell transplantation (allo-HSCT) for refractory neuroinflammation. The parents, A-I-1 and A-I-2, were clinically unaffected (**Figure 1A**).

In family B, the proband (B-II-2) first presented at the age of 5 weeks with fever, coryzal symptoms, severe anemia (Hb 64 g/dL) and splenomegaly (**Table 1**, **Figure 1B** ; **Supplementary Tables 1** and **2**). She was treated with IV antibiotics but pathogen screens were negative apart from molecular detection of *human herpesvirus 6* (HHV6) in whole blood, considered of dubious significance at that time. She suffered with anemia of unknown cause, exacerbated following immunizations or coryzal illnesses requiring repeated RBC transfusions. She had massive splenomegaly, persistently raised inflammatory markers, and transaminitis (ALT 343-977 U/L; RR 10-25 U/L; **Table 1**; **Supplementary Figure 1C**). Hemoglobinopathy screen, glucose-6-phosphate dehydrogenase, iron, folate and vitamin B12 levels were normal. Bone marrow showed megaloblastic and mildly dysplastic erythropoiesis.

Aged 4 years 3 months, she presented with reduced consciousness, seizures and gait abnormalities and was treated with high dose IV ceftriaxone and acyclovir for suspected (but unproven) infectious meningoencephalitis. She was afebrile but had elevated inflammatory markers (CRP 93 mg/L, ESR 50 mm/hr, SAA 343 mg/L) as well as low hemoglobin (64 g/L). She was therefore referred to our center for further assessment of suspected autoinflammation and neuroinflammation. She did not have any evidence of skin or lung pathology, and her eyes were normal. Immunology and autoimmune screens were normal/negative; high IL-6 levels were found in the CSF (107 pg/ml; RR<50) and blood ISG assay showed slightly elevated IFI27 (copy number threshold 4.68; RR 0.09 – 2.24; **Supplementary Table 3**). EEG showed continuous high amplitude irregular slow activity over the right hemisphere suggestive of right hemispheric dysfunction (**Supplementary Table 4**). A CT brain showed bitemporal cortical calcification and brain MRI showed chronic bilateral temporal cortical hyperintensities (**Table 1**; **Figure 1E**). Brain biopsy showed scattered inflammatory cells including perivascular T lymphocytes and diffuse upregulation of activated microglial cells and macrophages (**Figure 1F** and **Supplementary Table 4**). Extensive infective screens in blood, CSF, and from the brain biopsy (including metagenomic studies for infection) were negative for pathogens (**Supplementary Tables 3**, **4**). The patient was commenced on IV tocilizumab (12 mg/kg every 2 weeks, and anti-seizure medication (lamotrigine) with rapid and complete resolution of systemic autoinflammatory features including complete resolution of anemia and normalization of acute-phase reactants. Evidence of neuroinflammation persisted, however, on serial MRI brain scans with ongoing seizures. In addition, on tocilizumab, she had episodes of febrile neutropenia requiring changes to the dose (**Supplementary Table 2**). Over the course of several months, seizures persisted (3-6 times per week) and were particularly exacerbated by minor intercurrent respiratory infections. Consequently, tocilizumab was stopped and she was commenced on baricitinib (2 mg three times daily) and prednisolone 30 mg/day (1 mg/kg/day; **Supplementary Table 2**). However, it was impossible to wean the prednisolone below 0.5 mg/kg/day due to deterioration of acute-phase response markers (CRP 51 mg/L; ESR 25 mm/h; SAA not documented) and ongoing CNS inflammation. She also developed severe glucocorticoid toxicity with marked weight gain and severe behavioral disturbance. Therefore, at the time of writing she is being converted back to subcutaneous tocilizumab (162 mg [6.6 mg/kg] every 2 weeks) in combination with mycophenolate mofetil (600 mg/m^2^ twice daily) in an attempt to control ongoing neuroinflammation and allow glucocorticoid sparing; she is also being considered for allo- HSCT at the time of writing for refractory neuroinflammation.

The probands’ elder sister (B-II-1) presented with similar features from the age of 8 weeks with multiple episodes of fever, elevated inflammatory markers, massive splenomegaly and severe anemia requiring regular RBC transfusions. Episodes occurred in the absence of an infectious cause. She underwent splenectomy for ongoing transfusion dependent anemia of unknown cause aged 5 years, prior to referral to our institute. Post-splenectomy her Hb remained stable. However, on review at our center aged 10 years, she was found to have had persistently elevated inflammatory markers over the 5 years post-splenectomy (CRP 15 mg/L; ESR 90 mm/h; SAA 167 mg/L) and elevated IFI27 gene expression in peripheral blood interferon signature testing (**Table 1**; **Supplementary Tables 1**, **3**). Brain imaging was unremarkable with no evidence of past or present neuroinflammation (**Table 1**). At the time of writing, she has started subcutaneous tocilizumab 162 mg (4 mg/kg) weekly, with prompt normalization of acute-phase reactants after 2 weeks, and with annual MRI brain scanning to screen for possible future development of neuroinflammation.

B-II-3, the younger sister, had experienced intermittent fevers since the neonatal period but without anemia and on review at our center also had splenomegaly, elevated inflammatory markers and elevated IFI27 expression on peripheral blood interferon gene signature testing (111.13 - 395.87, RR 0.09 – 2.24; **Table 1**, **Figure 1B**). Like her siblings, she did not have any evidence of skin or lung pathology, and her eyes were normal. She had normal development and no clinical seizures, but neuroimaging revealed symmetrical signal abnormality within the medial temporal regions, the temporal stems, and in the white matter abutting the frontal horns (data not shown). She was therefore also commenced on baricitinib (2 mg three times daily; **Supplementary Table 2**), with prednisolone 1 mg per kilogram per day weaning over 6 weeks. However, 3 months after starting treatment, anemia had further deteriorated, and her inflammatory markers remained elevated (Hb 67 g/L; CRP 34 mg/L; SAA 47.4 mg/L; ESR 77). Thus, at the time of writing she has been converted to subcutaneous tocilizumab (162 mg [11 mg/kg] every 2 weeks) combined with mycophenolate mofetil (600 mg/m^2^ twice daily) to treat systemic inflammation and neuroinflammation, while also undergoing work up for potential allo-HSCT.

B-II-4 was clinically unaffected and with normal laboratory investigations (**Table 1**). The parents were clinically well but no laboratory testing for acute-phase response was performed.

### Identification of novel IRAK4 gene mutations

Whole exome sequencing of families A and B revealed mutations in *IRAK4*. In family A, both affected siblings A-II-1 and A-II-2 were found to be compound heterozygotes with variants in exons 8 (c.C877T) and 9 (c.G958T) of *IRAK4*, confirmed by Sanger sequencing (**Figures 1A, C** **)**. Both siblings inherited the previously described loss-of-function variant c.C877T (p.Q293*) from their father (A-I-1) and the novel variant, c.G958T (p.D320Y), from their mother (A-I-2). Both mutations are located within the kinase domain of *IRAK4* and the nonsense mutation p.Q293* has been shown to be deleterious, resulting in a premature stop codon and undetectable mRNA and protein expression (8). In family B, all affected siblings B-II-1, B-II-2, and B-II-3 were found to be compound heterozygous for two variants of *IRAK4*, c.A86C in exon 2 and c.161 + 1G>A (**Figures 1B, D** **)**. The c.A86C (p.Q29P) is a novel variant in the IRAK-4 DD, inherited from their father (B-I-1), and the c.161 + 1G>A variant is a novel variant inherited from their mother (B-I-2), predicted to be deleterious due to loss of donor splice site and resultant loss of protein expression (17). Similar non-coding variants affecting splicing have been previously described in *IRAK4* (28). B-II-4, who was clinically unaffected, was found to have inherited both wild type (WT) alleles of *IRAK4*.

### CD62L shedding is impaired (but not absent) in granulocytes from patients

**Figure 2:**
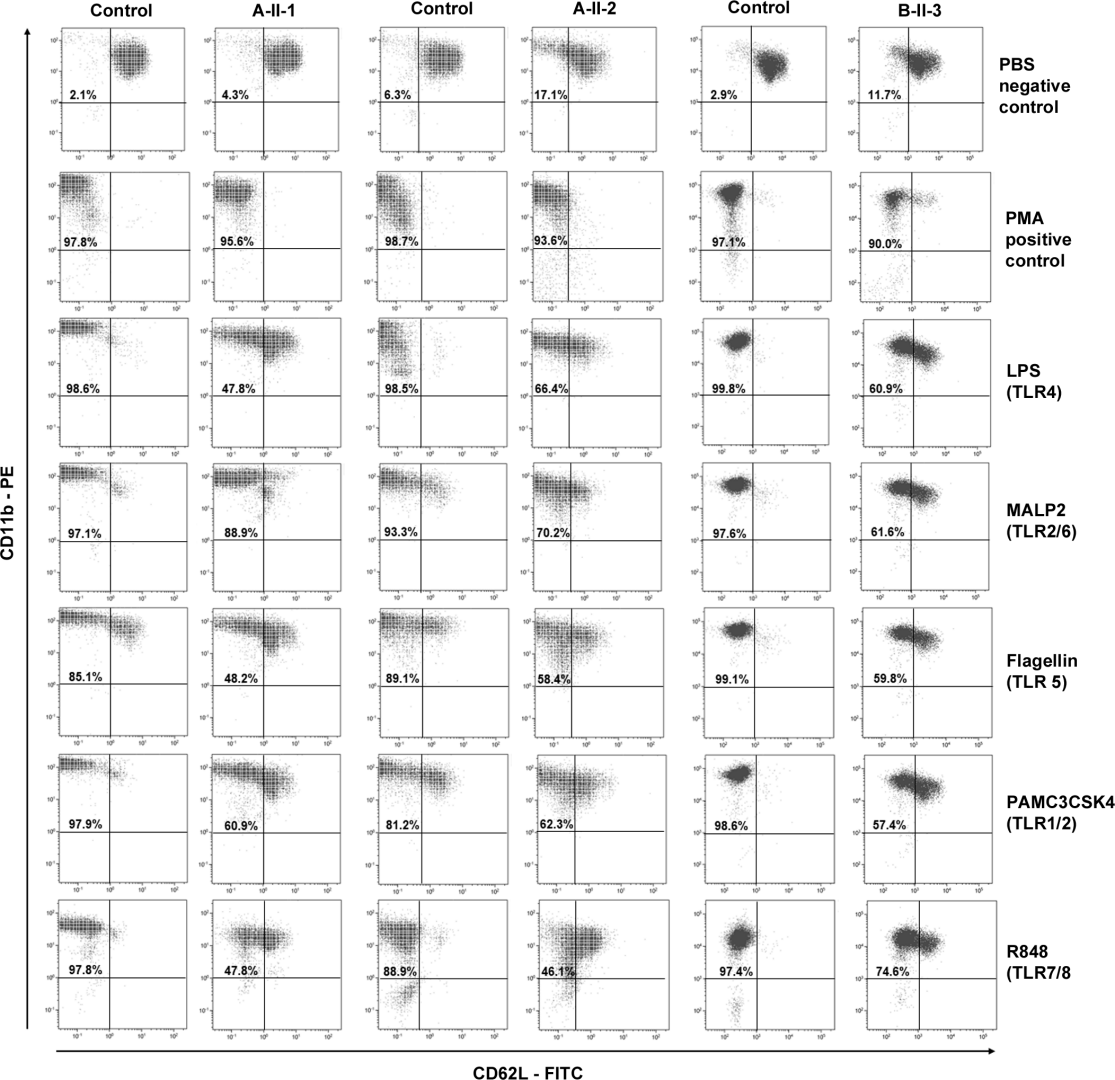
Shedding assay results. CD62L shedding was assessed by flow cytometry results for CD11b positive granulocytes from affected patients A-II-1, A-II-2, and B-II-3 and healthy adult controls following Toll-like receptor (TLR) agonists LPS (TL4), MALP-2 (TLR2/6), Flagellin (TLR5), PAM3CSK5 (TLR1/2) and R-848 (TLR7/8) stimulation. Phosphate buffered saline (PBS) and phorbolmyristyl acetate (PMA) were used as negative and positive controls, respectively. The percentage of gated granulocytes that have shed CD62L is indicated in the top left-hand quadrant.

Cleavage of CD62L (L-selectin) on granulocytes from patients upon stimulation with TLR agonists is severely impaired in patients with IRAK-4 deficiency (18). Therefore, CD62L shedding assays were carried out to test the function of IRAK-4 for family A and B-II-3 in family B. Shedding assays were conducted prior to immunosuppression in patients A-II-2 and B-II-3; patient A-II-1 was on tocilizumab at the time the assay was performed (which probably had no influence on the readout based on a previous study) (29). Viable samples were not available for testing shedding from other members of family B. Shedding of CD62L was found to be normal in granulocytes from heterozygotes, A-I-1 and A-I-2 (data not shown), with one wild-type copy of *IRAK4*, with normal loss of CD62L surface expression upon stimulation with LPS. Compound heterozygotes A-II-1, A-II-2 and B-II-3 showed partially reduced, but not absent, shedding of CD62L in response to LPS and other TLR agonists (**Figure 2**). A degree of shedding also occurred in unstimulated cells (PBS control) from A-II-2 and B-II-3. All patients and controls showed 90-97% shedding with the PMA positive control.

### Structural modeling predicts altered interaction of p.Q29P IRAK-4 DD (family B) with the DD of IRAK-1/2

**Figure 3:**
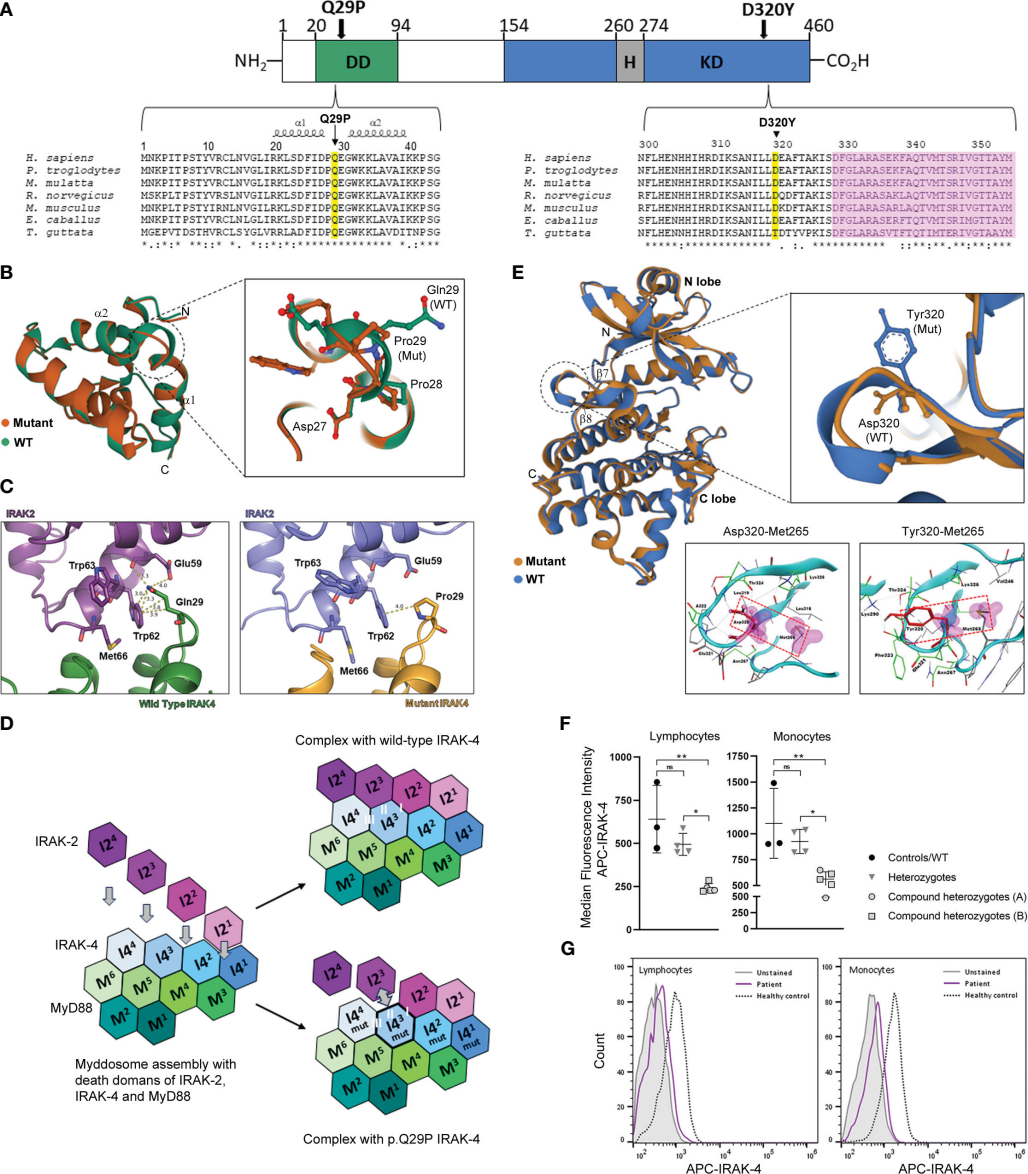
Location and structural modeling of IRAK-4 mutations and impact on protein expression. **(A)** Schematic representation of the 460 amino acid IRAK-4 protein showing the N-terminal death domain (DD; green), the C-terminal kinase domain (KD; blue) and within the KD domain the hinge region (H; grey). The D320Y and Q29P mutations identified in family A and family B, respectively are indicated by black arrows. Multiple sequence alignment of IRAK-4 amino acid sequences surrounding Q29 within the DD and D320 within KD of IRAK-4 from different species (yellow). The location of α-helices 1 and 2 and the activation segment (pink) are also indicated. **(B)** Superposition of mutant Pro29 and wild type Gln29 between α-helices 1 and 2 (α1 and α2) in the structure of the death domain of IRAK-4. Adjacent residues Asp27 and Pro28 are also shown. **(C)** Analysis of the interface between p.Q29P IRAK-4 and IRAK-2, compared to wild type IRAK-4, showing increased distance with loss of hydrogen bond and hydrophobic interactions. **(D)** Schematic representation of myddosome assembly of DD of MyD88 (M^1^-M^6^), IRAK-4 (I4^1^-I4^4^), and IRAK-2 (I2^1^-I2^4^) and the predicted impact of the p.Q29P on the interaction between IRAK-4 (I4mut^3^) and IRAK-2 (I2^3^) and interacting interface II of I-III. **(E)** Superposition of mutant Tyr320 and wild type Asp320 located in the loop region between β-sheets 6 and 7 (β6 and β7) in the kinase domain structure of IRAK-4. Analysis of interaction of atoms of Asp320 and mutant Tyr320 residues were examined within a radius of 4.5 Å using a protein contacts panel, showing hydrophobic interaction with Met265. **(F)** Flow cytometry analysis of intracellular IRAK-4 expression in patient derived PBMCs. Healthy controls (black circles) and wild type patient (B-II-4; lower circle), heterozygous parents (grey inverted triangles) and compound heterozygotes from family A (grey circles) and family B (grey squares) are shown. **(G)** Representative flow cytometry histograms of IRAK-4 expression (median fluorescence intensity APC, allophycocyanin) in lymphocytes and monocytes from an affected patient (purple) versus a healthy control (dashed) are shown compared to unstained cells (grey). Statistical significance is indicated by p-values (*0.01 and** 0.001 in lymphocytes and *0.03 and **0.007 in monocytes), with non-significance indicated by ns.

IRAK-4 is a 460 amino acid protein with a structure that consists of an N-terminal DD that interacts with the DD of MyD88 and IRAK1/2 within the myddosome complex and a C-terminal kinase domain (KD) that catalyzes autophosphorylation, and *trans*-phosphorylation and activation of downstream substrates (**Figure 3A**). The p.Q29P mutation in family B is located within an evolutionarily conserved region of the IRAK-4 DD between α-helices 1 and 2 (**Figure 3A**) (30). The Q29 residue has been shown to be structurally important for the interdigitation of the interface between IRAK-4 and IRAK-2 and helical assembly of the myddosome complex (5). Structural modeling and superposition of a non-polar proline residue (p.Q29P; Pro29 mutation) onto the DD of IRAK-4 compared to the wild-type polar glutamine (Gln29) demonstrates a movement of the loop connecting α-helices 1 and 2 as well as movement of Pro28 and Glu30 residues (**Figure 3B**). Modeling of IRAK-4 DD Pro29 in place of Gln29 at the DD binding interface of IRAK-4 and IRAK-2 [PDB: 3MOP] demonstrated an increase in contact distance between residues with loss of hydrogen bond interactions with Glu59 and the majority of hydrophobic interactions with Trp62 within IRAK-2 (**Figure 3C**). The myddosome complex forms from sequential assembly of the death domains of six MyD88 (M^1^-M^6^) proteins, four IRAK-4 (I4^1^-I4^4^) proteins and four IRAK-2 (I2^1^-I2^4^) proteins (**Figure 3D**) (5). Within the myddosome complex, this increased distance and loss of contacts due to the Pro29 mutation is predicted to affect the interaction of the third molecule of IRAK-4 (I4^3^) with the third molecule of IRAK-2 (I2^3^) during myddosome assembly, referred to as interacting surface ‘II’ out of I-III by Lin et al. (**Figure 3D**) (5). The functional impact of this is not known but may lead to instability and aggregation or disordered assembly resulting in dysregulated downstream signaling.

### Structural modeling of p.D320Y IRAK-4 (family A) predicts stabilization of the kinase domain active site

The Tyr320 mutated residue (p.D320Y) from family A is located at an evolutionarily conserved region of the IRAK-4 KD, adjacent to the activation segment (**Figure 3A**). The KD consists of an N-terminal lobe with a 5-stranded, antiparallel β-sheet and a helix, as well as an α-helical C-terminal lobe (**Figure 3E**) (24, 30). Activation of IRAK-4 depends on *trans*- and autophosphorylation of Tyr345 within a kinase activation loop. The ATP active site lies between the two lobes and a hinge region connecting the lobes is thought to be important for conformational changes that regulate ATP binding and enzyme activation (31). Modeling of the Tyr320 mutation onto the crystal structure of IRAK-4 [PDB: 2NRU] in place of Asp320 shows that the bulky tyrosine residue alters the orientation of the loop between β-sheets 7 and 8 (**Figure 3E**) (30). A protein contacts panel shows that this change in orientation would allow interaction with residue Met265 within the hinge region that contributes to the hydrophobic binding pocket crucial for binding ATP, a prerequisite for kinase autophosphorylation and IRAK-4 activation (**Figure 3E** and **Supplementary Tables 5**, **6**). As such it would be predicted to have a stabilizing effect on the IRAK-4 kinase active site and potentially maintain IRAK-4 in active state. Modeling of different IRAK-4 inhibitors into the active site of the IRAK-4 KD in the presence of Tyr320 has been shown to negatively impact inhibitor binding and decrease conformational stability of the protein (**Supplementary Results** and **Supplementary Figures 3**–**5**).

### IRAK-4 protein expression is reduced but not absent in patient PBMC

Analysis of IRAK-4 protein expression by flow cytometry in lymphocytes and monocytes derived from PBMC, demonstrated that affected compound heterozygotes (A-II-1, A-II-2, B-II-1, B-II-2 and B-II-3) had reduced but not absent IRAK-4 expression compared to healthy controls (lymphocytes, p=0.001; monocytes, p=0.007; **Figure 3F**) and unaffected carriers (lymphocytes, p=0.01; monocytes, p=0.03). Unaffected carriers (A-I-1, A-I-2, B-I-1 and B-I-2), with one wild-type copy of *IRAK-4* and B-II-4 with two wild-type alleles had normal levels of IRAK-4 expression (**Figure 3F** **).** Residual expression is likely to be a result from the p.D320Y (family A) and p.Q29P (family B) mutant IRAK-4 proteins as p.Q293* has been shown to be deleterious and c.161 + 1G>A (splice site) is predicted to be deleterious from *in silico* modeling. Representative flow cytometry histograms of IRAK-4 APC expression in lymphocytes and monocytes from an affected patient and a healthy control is shown in **Figure 3G**.

### Multiple cytokine levels are elevated in serum of patients, but only IL-6 is elevated in CSF

**Figure 4:**
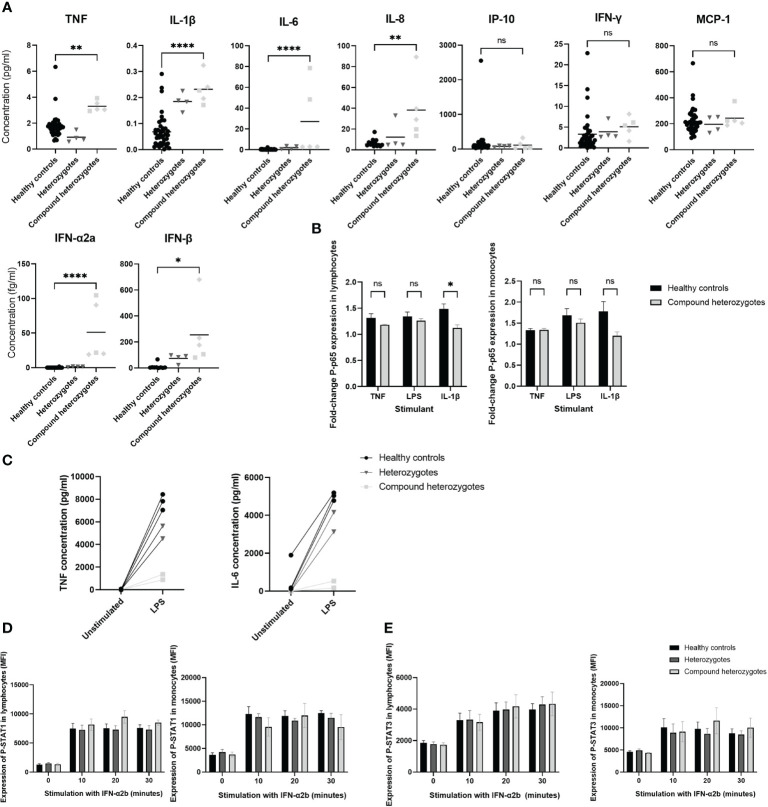
Functional evaluation of IRAK-4 signaling. **(A)** Pro-inflammatory cytokine quantification in sera using Meso Scale Discovery. Healthy controls (black circles), heterozygote parents (dark grey inverted triangles) and compound heterozygotes from Family A (light grey squares) and Family B (light grey diamonds), are shown. **(B)** NF-κB signaling assessed via flow cytometry analysis of phosphorylated-p65 (P-p65) stimulated with ligands as indicated on x-axis, fold-change median fluorescence (MFI) from baseline at 20 minutes shown. **(C)** Analysis of interleukin-6 (IL-6) and tumor necrosis factor (TNF) production by peripheral blood mononuclear cells (PBMC) from Family A in response to lipopolysaccharide (LPS) stimulation. **(D)** STAT1 and **(E)** STAT3 signaling assessed via flow cytometry analysis of phosphorylated-STAT1 (p-STAT1) and STAT3 (p-STAT3) expression following stimulation with interferon α2b (IFN-α2b) at time points indicated on x axis. Horizontal lines at mean and standard error (SE) of the mean shown. Significance indicated by: *p < 0.05; ** p < 0.01; and ****p < 0.0001 and ns, not significant. TNF, tumor necrosis factor; IL, interleukin; IP-10, interferon-γ induced protein-10; MCP-1, monocyte chemoattractant protein-1; IFN, interferon; LPS, lipopolysaccharide; p-STAT1/3, phosphorylated signal transducer and activator of transcription 1/3; MFI, median fluorescence intensity.

Levels of pro-inflammatory cytokines in sera were increased in affected *IRAK4* compound heterozygotes compared to controls: TNF (p ≤ 0.01); IL-1β (p ≤ 0.0001); IL-6 (p ≤ 0.0001); IL-8 (p ≤ 0.01); IFN-α2a (p ≤ 0.0001); and IFN-β (p ≤ 0.05), while there was no significant difference observed in levels of MCP-10 (p = 0.77); IP-10 (p = 0.86); and IFN-γ (p = 0.64) (**Figure 4A**). CSF IL-6 levels were elevated in both children who underwent lumbar puncture: A-II-2 IL-6 (225 pg/ml, RR <50; taken while on tocilizumab); and B-II-2 IL-6 (107 pg/ml, RR<50; taken prior to starting tocilizumab). No other CSF cytokine was found to be elevated in either patient (**Supplementary Table 3**).

### Patient-derived PBMC demonstrate normal NF-κB signaling but reduced cytokine response to LPS stimulation

Downstream of TLRs (excluding TLR3), IRAK-4 predominantly signals via NF-κB (2). Therefore, we assessed levels of phosphorylated NF-κB phosphorylated p65 (P-p65) in PBMC derived from affected *IRAK4* compound heterozygote patients A-II-1, A-II-2 and B-II-2. P-p65 level was unchanged at baseline compared to healthy control cells (lymphocytes, p=0.69; monocytes, p=0.95), and there was no difference between patients and controls in the fold increase in P-p65 following a 20 minute stimulation with TNF or LPS (**Figure 4B** and **Supplementary Figure 2**). However, there was reduced P-p65 response to IL-1β in lymphocytes (p ≤ 0.05); and a similar trend for monocytes, although not reaching statistical significance (**Figure 4B**).

In PBMC from family A, following a 6-hour stimulation with LPS there was reduced production of cytokines IL-6 (p ≤ 0.01) and TNF (p ≤ 0.001) in affected compound heterozygotes compared to heterozygotes or healthy controls (**Figure 4C**). Insufficient samples were available to replicate this experiment in family B.

### Patient-derived PBMC demonstrate normal STAT1/3 signaling

Since we demonstrated raised type 1 interferons in serum (**Figure 4A**) and perturbation of peripheral blood interferon-stimulated gene signature (**Supplementary Table 3**) in the context of neuroinflammation and intracerebral calcification, we explored whether STAT signaling was upregulated. Therefore, we assessed expression of phosphorylated STAT1 in PBMC derived from patients A-II-1, A-II-2 and B-II-2, and found this to be unchanged compared with healthy control cells both at baseline (lymphocytes, p=0.96; monocytes, p=0.99) and in response to stimulation with IFN-α2b (lymphocytes, p=0.45; monocytes, p=0.65) (**Figure 4D**). Similarly, expression of phosphorylated STAT3 in patients compared with healthy controls was unchanged both at baseline (lymphocytes, p=0.79; monocytes, p=0.55) and in response to stimulation with IFN-α2b (lymphocytes, p=0.98; monocytes, p=0.94) (**Figure 4E**). Taken together these results indicated that the mutations in *IRAK4* were not primarily driving a downstream type I interferon response via upregulation of STAT1 or STAT3.

## Discussion

We describe a novel, highly penetrant, and severe autoinflammatory disease caused by bi-allelic mutation in *IRAK4*. The clinical features are summarized by the acronym NASA: neuroinflammation, systemic autoinflammation, transfusion dependent anemia, and massive splenomegaly in the absence of severe immunodeficiency. Affected individuals were phenotypically similar, although at the time of writing 2 out of 5 of the affected cases did not have evidence of neuroinflammation. Longer term follow-up will determine if this feature develops over time in these cases. Blockade of IL-6 has proven highly effective for systemic autoinflammation and anemia but has failed to control neuroinflammation in the 3 cases with this complication. Janus kinase inhibition with baricitinib failed to control systemic autoinflammation and neuroinflammation in 3 of the cases. IL-1 blockade had no effect on the proband of family A. Our attempts to define the mechanism of autoinflammation point away from a primary type I interferonopathy but do suggest a role for autoinflammation driven by IL-6, notably with high IL-6 in the CSF of 2 affected patients with severe neuroinflammation. We did not find excessive activation of P65 in PBMCs from affected individuals that would indicate excessive NF-κB activation as the cause of autoinflammation in NASA; nor did we find perturbation of STAT1/3 activation. At face value, partly impaired CD62L shedding might contribute to autoinflammation, but this is unlikely to be the primary mechanism since autoinflammation has never been described in patients with typical IRAK-4 deficiency, who have severely impaired shedding. The cause of autoinflammation in NASA therefore remains uncertain but seems to be driven predominantly by IL-6 systemically, and in the CNS.

The novel *IRAK4* mutations described in this study were located in different domains of the IRAK-4 protein: p.Q29P (family B) in the DD; and p.D320Y (family A) in the KD yet resulted in identical clinical phenotypes. The p.Q29P mutation alters the distance and hydrophobic binding interactions with IRAK-2 within the myddosome complex (5). We postulate that this mutation could therefore disrupt the regular helical assembly of the myddosome complex in response to TLR stimulation, resulting in an aggregated protein structure, disordered assembly or disassembly, and dysregulation of IRAK-1/2 and IRAK-4 signaling. Structural modeling of the p.D320Y (family A) within the kinase domain activate site of IRAK-4 predicted that it would stabilize the active site allowing for autophosphorylation and constitutive activation. However, the presence of a bulky tyrosine residue prevents binding of larger inhibitory molecules within the active site of IRAK-4. The net effect of this would be dysregulated and constitutively activated IRAK-4 driving autoinflammation in compound heterozygote patients with one null, and one missense mutation.

In contrast to patients with IRAK-4 deficiency, reduced, but not absent IRAK-4 expression was observed in PBMC of patients with NASA. Furthermore, our cases had normal lymphocyte subsets and vaccine responses and did not experience pyogenic bacterial infections. B-II-1 and B-II-2 were found on one occasion each to have detectable HHV-6 DNA in blood in the neonatal period, and reactivation of HHV-6 has been reported in the context of IRAK-4 deficiency (32, 33). However, this was likely not specific to their clinical phenotype as HHV-6 reactivation is commonly observed in healthy babies and children during periods of acute illness (34). Other pathogens were not identified despite extensive screening.

A number of studies have described neuroinflammation in patients with IRAK-4 deficiency in the context of presumed infective/autoimmune encephalitis. Our data could suggest that neuroinflammation in IRAK-4 deficiency may not always be due to infection, but rather as an inherent autoinflammatory feature caused by perturbation of this important signaling pathway. Indeed, the presence of severe neuroinflammation in our cases in the absence of infection implies that fully functional IRAK-4 likely plays an important role in the regulation of the inflammatory milieu within the CNS (33, 35, 36). Of note, B-II-3, had no clinical neurological features, but was found on screening to have extensive changes on brain imaging. We, therefore, suggest that screening for neuroinflammation should be undertaken in all patients with bi-allelic *IRAK4* mutations, and that regular serial monitoring for this complication using MRI brain be undertaken, even in the absence of overt neurological symptoms.

Systemic inflammation in our patients was evidenced by persistently elevated CRP, ESR and serum amyloid A. This contrasts with the profile seen in patients with classic autosomal recessive IRAK-4 deficiency, who are typically slow to mount an inflammatory response, often with lower inflammatory markers, even in episodes of bacteremia (27). We further characterized cytokine dysregulation and observed elevated levels of circulating TNF, IL-1β, IL-8, IFN-α2a and IFN-β. Although serum IL-6 was highly raised in A-II-1 and A-II-2, both were on tocilizumab at the time of sampling, making this difficult to interpret in those patients (37, 38). High IL-6 was also observed in the CSF of A-II-2, again after starting tocilizumab. However, the high level of IL-6 observed in the CSF of B-II-2 is not the result of tocilizumab since this was measured prior commencing this treatment. Taken together, these observations strongly suggest that neuroinflammation in NASA is driven by IL-6.

A seemingly discordant observation was that stimulation of patient PBMC with LPS resulted in reduced levels of IL-6 and TNF production compared with controls. This emphasizes that there is functional impairment of the IRAK-4 pathway in patients with NASA, but that autoinflammation rather than immune deficiency predominates the clinical picture. We cannot, at the moment, conclude that the autoinflammation is the direct result of gain-of-function (GOF) for the novel variants we have described, but rather reflects loss of normal physiological regulation. Although modeling of the p.D320Y suggests stabilization of the kinase domain, arguing against true GOF are: 1. the mother (A-I-2) in family A had no phenotype or immunophenotype (including normal CD62L-shedding) despite carrying this variant; 2. protein expression was reduced; and 3. we observed reduced cytokine responses to LPS stimulation *in vitro*. As IRAK-4 can autophosphorylate itself, it would be necessary to express the protein and crystallize it in the presence of the p.D320Y mutation to better understand if this increased or decreased protein stability and therefore autophosphorylation and activation. Crystallization of the p.Q29P DD of IRAK-4 with the DD of IRAK-2 and MyD88 would be required to ascertain the true impact on the helical oligomeric assembly of the myddosome complex. NASA patients could still have a degree of functional innate immune deficiency in response to PAMP triggers, which should be borne in mind when using anti-cytokine directed treatments. Therefore, NASA probably joins a growing group of inborn errors of immunity spanning autoinflammation and immune deficiency.

IL-6 receptor blockade with tocilizumab was remarkably efficacious at amelioration of systemic autoinflammation and transfusion dependent anemia in affected individuals. Tocilizumab did not ameliorate neuroinflammation, however, probably due to poor CNS penetration of this drug, an issue common to therapeutic IgG biologic therapies (39). Although the CSF pharmacokinetics of JAK inhibitors have not been fully elucidated, recent studies in humans have also suggested relatively poor CNS penetration (levels 10-15% of serum) for both ruxolitinib and baricitinib (37, 38), despite animal studies demonstrating its ability to cross the blood-brain barrier (40). This might explain why JAK inhibitors have limited efficacy for neuroinflammation in other autoinflammatory disorders and monogenic interferonopathies with neurological involvement, such as Aicardi–Goutières syndrome (41–43). Indeed, a three-month therapeutic trial of baricitinib (using a monogenic interferonopathy dosing schedule) (44) in 3 of the cases failed to control neuroinflammation (and in fact also failed to control systemic autoinflammation, and anemia). Therefore, at the time of writing, these 3 patients have been converted back to tocilizumab, with the addition of an alternative immunosuppressive agent, mycophenolate mofetil which has a track record for treating other forms of neuroinflammation such as CNS vasculitis (45). Time will tell if this proves effective or not; for that reason, all 3 cases with refractory neuroinflammation are being considered for allo-HSCT although to the best of our knowledge, this has never been performed in any patient with IRAK-4 deficiency. An alternative could be TNF blockade, although arguably the size of this molecule would also limit its penetration across the blood-brain barrier. Intrathecal tocilizumab might be an option, as described in 2 cases of graft-versus-host disease (46), but is not viable or practical in the long-term.

The anemia observed in NASA patients almost certainly is due to severe chronic systemic autoinflammation rather than immune-mediated mechanisms (as occurs in patients with *TLR8* mutations causing TLR7-mediated autoinflammation) (14). Pro-inflammatory cytokines such as IL-6, IL-1 and TNF restrict erythropoiesis. Consistent with this the bone marrow of patients A-II-2, B-II-1 and B-II-2 exhibited activated erythrocytes and expanded dysplastic erythropoiesis. The rapid resolution of anemia following treatment with tocilizumab further supports this mechanism. Notably, however, anemia in B-II-2 partially ameliorated following splenectomy, although systemic inflammation persisted, suggesting that secondary hypersplenism may also play a role. We do not, however, advocate for splenectomy in patients with NASA.

Our study is limited by the availability of patient material and reagents to assess phosphorylated IRAK-4, IRAK-1 and other phosphorylated components of the IRAK-4 pathway. Hypomorphic *IRAK4* mutations could lead to autoinflammation via loss of negative regulation of itself and also of IRAK-1 (47). Levels of phosphorylated IRAK-4 and -1 in patient cells were too low to study using commercially available anti-phospho-antibodies by Western blotting; moreover, no good quality antibodies were available for flow cytometry. Further functional studies are required to understand the exact mechanism of IRAK-4 signaling that results in autoinflammation. A further limitation is that we were not able to have access to an assay to directly measure tocilizumab levels in the CSF, so can only assume that the lack of therapeutic response regarding neuroinflammation relates to poor CNS penetration in the face of high CSF IL-6 levels.

In conclusion, we describe a novel and severe monogenetic autoinflammatory disease. Affected individuals had compound heterozygous mutations in *IRAK4* where one allele was a loss-of-function variant and the other a novel variant resulting in reduced, but not absent, protein expression and abnormal CD62L-shedding, and excess of proinflammatory cytokines, including high CSF IL-6 levels. We propose the name NASA (neuroinflammation, autoinflammation, splenomegaly and anemia) for this new autoinflammatory disease, and emphasize that clinicians should consider each component of the acronym separately since treatment with IL-6 blockade so far has proven extremely efficacious for systemic autoinflammation and transfusion dependent anemia but has not had any impact on the neuroinflammation or massive splenomegaly. In particular, we suggest that screening for neuroinflammation with brain MRI should be undertaken in all patients with bi-allelic mutations in *IRAK4* and also hypothesize than autoinflammation may be more prevalent that hitherto recognized in patients with typical IRAK-4 immunodeficiency.

## Data availability statement

Sequencing data are available for family A in the NCBI BioSample database (http://www.ncbi.nlm.nih.gov/biosample/) under accession numbers: SAMN35661569, SAMN35661570, SAMN35661571, SAMN35661572. Family B did not specifically consent to public sharing of their whole exome sequencing data. Requests to access the datasets should be directed to VS (v.sancho-shimizu@imperial.ac.uk).

## Ethics statement

The studies involving humans were approved by Great Ormond Street Hospital NHS Foundation Trust (GOSH) Research Ethics Committee. The studies were conducted in accordance with the local legislation and institutional requirements. Written informed consent for participation in this study was provided by the participants’ legal guardians/next of kin.

## Author contributions

SCo and PB conceptualized the study; SCo, FP-K, and PB drafted the manuscript; SCo, FP-K, EO, YH, DE, BJ, KG, AG and AB collected and analyzed the data and SCo, FP-K, EO, YH, AB, KG, AG, DE, SCh, BA, and MWB contributed to methods and results. BA, SCh, SCo, and MB provided detailed structural modeling and analysis. EB and VS provided genetic results for family B. EW, AW, JH, PT, JH, KM, MK, and AK provided detailed clinical information. All authors contributed to the article and approved the submitted version.
