# Supplementary material for: Neuroinflammation, autoinflammation, splenomegaly and anemia caused by bi-allelic mutations in IRAK4
Source: Front Immunol. 2023 Sep 6;14:1231749. doi: 10.3389/fimmu.2023.1231749 (PMC10516541; doi:10.3389/fimmu.2023.1231749)
Supplement: Supplementary file 1 [file DataSheet_1.docx]

Neuroinflammation, autoinflammation, splenomegaly and anemia (NASA) caused by bi-allelic mutations in *IRAK4*.

Samantha Cooray^1*,^ Fiona Price-Kuehne^1^, Ying Hong^1^, Ebun Omoyinmi^1^, Alice Burleigh^1,2^, Kimberly Gilmour^3^, Bilal Ahmed^4^, Sangdun Choi^4^, Mohammed W. Bahar^5^, Paul Torpiano^3^, Andrey Gagunashvili^6^, Barbara Jensen^1^, Evangellos Bellos^7,8^, Vanessa Sancho Shimizu^7,8^, Jethro Herberg^7,9^, Kshitij Mankad^10^, Atul Kumar^11^, Marios Kaliakatsos^12^, Austen J. J. Worth^3^, Despina Eleftheriou^1^, Elizabeth Whittaker^7,9^, and Paul Brogan^1^.

^1^Infection, Immunity and Inflammation Department, University College London Great Ormond Street Institute of Child Health, London, UK;

^2^Centre for Adolescent Rheumatology Versus Arthritis, University College London, London, UK;

^3^Department of Immunology and Gene Therapy, Great Ormond Street Hospital for children NHS Foundation Trust, London, UK;

^4^Department of Molecular Science and Technology, Ajou University, Suwon 16499, Korea;

^5^Division of Structural Biology, University of Oxford, The Henry Wellcome Building for Genomic Medicine, Oxford, UK;

^6^Faculty of Life and Environmental Sciences, University of Iceland, Sturlugata 7, Reykjavík, 101, Iceland;

^7^Section of Paediatric Infectious Diseases, Imperial College London, London, UK;

^8^Centre for Paediatrics and Child Health, Faculty of Medicine, Imperial College London, London, UK;

^9^Department of Paediatric Infectious Diseases, St Mary's Hospital, Imperial College NHS Healthcare Trust, London, UK;

^10^Department of Radiology, Great Ormond Street Hospital for children NHS Foundation Trust, London, UK;

^11^Department of Histopathology, Great Ormond Street Hospital for children NHS Foundation Trust, London, UK;

^12^Department of Neurology, Great Ormond Street Hospital for children NHS Foundation Trust, London, UK.

*** Correspondence:**Corresponding Author
[Samantha.Cooray1@nhs.net](mailto:Samantha.Cooray1@nhs.net)

**Supplementary Methods**

**Structure preparation and molecular docking**

The structure of IRAK4 was retrieved from the RCSB Protein Data Bank (PDB ID: 5UIU). The D320Y mutant of IRAK4 was constructed using PyRosetta^1^ as in our previous protocol^2^. The molecular coupling of four inhibitors emavusertib, zimlovisertib, Bay-830839, and GS5718 was performed in (Molecular Operating Environment (MOE) Software as previously described^3^ by selecting the binding site of the ligand of the cocrystal. The final selected poses were subjected to molecular dynamics study for further analysis.

**Molecular dynamics (MD) simulations and trajectory analysis**

Molecular dynamics simulations of the IRAK4 protein and its D320Y mutant with inhibitors were performed using the charmm36 force field^4^ with GROMACS 2022.2^5^. The inhibitor topology was generated on the CHARMM general force field (CGenFF) server as described in our previous study^3^. After protein and inhibitor topology generation, the IRAK4-inhibitor system was solvated in a cubic box of explicit TIP3P^6^ water and neutralized with sodium ions. The energy was minimized by steepest descent minimization for each solvated and neutralized system. Each system was equilibrated for 1 ns under the NPT ensemble, with the pressure coupled to 1 atm with the Berendsen barostat^7^ and the temperature set to 310 K with the velocity-rescaling thermostat^8^. Heavy atoms were restrained in their positions (force of 1000 kJ mol-1 nm-1) during the equilibration. A simulation of 200 ns was performed for each system in the NVT ensemble with periodic boundary conditions after the system had been equilibrated. A 10 Å cutoff was used for van der Waals and short-range electrostatic interactions. Particle-Mesh Ewald (PME) summation was used for long-range electrostatic interactions^9^. Verlet cut-off scheme was used^10^. By using the LINCS algorithm^11^, we constrained the covalent bonds. The integration time-step was 2 fs. Data analysis was performed using Gromacs' built-in tools for the calculation of root mean square deviation (RMSD), PyMOL^12^ for visualization, and MdTraj^13^ with scikit-learn^14^ for principal component analysis (PCA).

**End-state binding free energy calculations**

To calculate IRAK4 inhibitor end-state free energy MM/PB(GB)SA (molecular mechanics/Poisson–Boltzmann (Generalized-Born) surface area) approach was used. Python based gmx_MMPBSA^15^ program was used to perform end-state free energy calculations. In accordance with the previous report^2,16^, the frames were extracted at intervals of 30 frames from the 200ns trajectory. The binding free energy for the protein and ligand was calculated using Eq. 1:

∆G_bind_= (G_complex_) - (G_protein_+ G_ligand_) (1)

ΔG_bind_ = ΔH - TΔS, (2)

where ΔH corresponds to the enthalpy of binding and −TΔS to the conformational entropy after ligand binding. The ΔH can be decomposed into different terms.

ΔH = ΔE_MM_ + ΔG_sol_ (3)

ΔE_MM_ represents the molecular mechanical energy changes in the gas phase and ΔGsol is the solvation energy difference in the above equation. Among the EMM components, the bonded components, also known as the internal energy, and the nonbonded components, corresponding to the van der Waals and electrostatic contributions. The entropic term (ΔS) was calculated using the interaction entropy method in the gmx_MMPBSA program.

**Supplementary Results**

**Dynamics of IRAK-4 inhibitor complexes in in the presence and absence of the p.D320Y mutation**

The RMSD profile and PCA of the IRAK-4 inhibitor were examined in the presence and absence of the p.D320Y mutation (**Supplementary Figure 3**)^17,18^. The RMSD trajectories showed a marked deviation in the p.D320Y mutant of the IRAK-4-zimlovisertib and IRAK-4-emavusertib complexes (**Supplementary Figure 3A I and 3B I**), while there was a slight change in RMSD of the complexes IRAK-4-Bay830839 and IRAK4-GS5718 (**Figure 3C I and 3D I**) compared to the corresponding wild types. The backbone RMSD curve of the p.D320Y mutant of IRAK4-zimlovisertib indicated that the system fluctuated from 0 to 30 Å whereas IRAK-4-zimlovisertib wild type remains within 20 Å (**Supplementary Figure 3A I**). It is noteworthy that the IRAK-4-emavusertib p.D320Y mutant showed a continuous upward trend of deviation throughout the simulation time period, with RMSD starting at 10 and reaching 40, while the wild type of IRAK4-emavusertib oscillates within 10 (**Supplementary Figure 3B I**). However, the RMSD curves of IRAK-4-Bay1830839 both wild-type and D320Y mutants showed a similar trend with increasing fluctuation of RMSD from 0 to 45 (**Supplementary Figure 3C I**). It is noticeable that after 125 ns of RMSD trajectory, the D320Y mutant of IRAK4-GS5718 shows a deviation from 15 to 20 ns (**Supplementary Figure 3D I**).

To analyze conformational changes and structural evolution along the MD simulation trajectory, PCA was used to extract the predominant motions of wild type IRAK-4 and the p.D320Y mutant in a complex with four inhibitors. The PCA of the wild-type -IRAK-4 and p.D320Y mutant IRAK-4-inhibitor complexes is shown, with continuous color representations from blue to yellow to indicate conformational changes. Blue and yellow were the most predominant subspaces of conformers in the wild-type IRAK-4 inhibitor complex. The p.D320Y IRAK-4-inhibitor complex showed periodic conformational switches. In contrast to the wild-type inhibitor-IRAK-4 complex, the p.D320Y mutant-inhibitor complexes dispersed widely and covered a large subspace (**Supplementary Figure 3A** **II, 3B II, 3C II)**. There is a wider dispersion of the conformational subspace of the IRAK-4 p.D320Y-Bay1830839 than with wild type IRAK-4, despite similar RMSD curves. PCA subspace analysis of IRAK-4-p.D320Y-GS5718 complex showed a similar pattern to zimlovisertib (**Supplementary Figure 3D II**). PCA data suggested that the p.D320Y mutation of IRAK-4 leads to conformational dispersion of the system. A continuous periodic jump from one state to another was evident in the graph, suggesting instability of IRAK-inhibitor complex formation in the presence of the p.D320Y mutation.

**Impact of the IRAK-4 p.D320Y mutation on inhibitor binding**

To obtain a more in-depth understanding of the impact of p.D320Y on inhibitor binding, we extracted the snapshots of trajectories at different time points. Superimposition of snapshots of the protein-ligand complex in the presence of p.D320Y mutation demonstrates what geometric changes are taking place in the interacting residues during simulation compared to wild type IRAK-4 (**Supplementary Figure 4**). The outward movement of the inhibitors appears to be coupled with conformational changes in IRAK-4 due to the p.D320Y mutation. Observation of the movement of inhibitor binding site residues E233, Y262, M265, D329 and F330 residues^19,20^ appears to show that the M265 residue of the hinge region important for inhibitor binding^19^ is further away from most inhibitors in the presence of the p.D320Y mutation, as visualized at 25, 75, 125 and 175 ns of simulation (**Supplementary Figure 4**). With the exception of GS5718, all other inhibitors were further away from the gatekeeper Y262 residue. Unlike GS5718, all other inhibitors (zimlovisertib, emavusertib and Bay1830839) lose contact with binding site residues due to conformational change caused by the p.D320Y mutation. It should be noted that zimlovisertib was still able to inhibit lipopolysaccharide (LPS) induced stimulation of IL-6 and TNFα in patient peripheral blood mononuclear cells (PBMCs), suggesting binding can still occur (data not shown, available on request).

**Influence of D320Y mutation of the IRAK4 on binding affinity of inhibitors**

We next examined the effect of the p.D320Y IRAK-4 mutation the on binding affanity of inhibitors. The p.D320Y mutation reduced the binding affinity of zimlovisertib, emavusertib, Bay830839 and GS5718 by -3, -2, -4, and -5 kcal/mol, respectively (**Supplementary Figure 5).** The total binding free energy diminished by 2–5% across each mutant system, confirming the decreased affinity of the inhibitors. The decrease in binding energy of the inhibitors due to the p.D320Y mutation occurred because the D320 residue is close to the activation loop (329–358)^20^. The p.D320Y mutation causes a conformational change in IRAK-4 as revealed by our PCA and RMSD data (**Supplementary Figure 4**). A change in the geometry of interacting residues caused by conformational change disrupts the inhibitor residue interaction. Changing the complex's conformation may lead to its dissociation ^21^.

**Supplementary Figures & Tables**

**Supplementary Figure 1.**

**Biochemical response to biologic treatment.** Trend of Hemoglobin (Hb; Reference range 115-145 g/L), C-reactive protein (CRP; Reference range <20 mg/L) and alanine aminotransferase (ALT; Reference Range 5-40 Units/L) values shown for affected patients A) A-II-1, B) A-II-2 and C) B-II-2 during the clinical course of illness, with shaded areas indicating commencement and period of treatment with anti-IL1 agents anakinra (purple) and canakinumab (blue), anti-IL6-receptor agent tocilizumab (green) and JAK1/2 inhibitor, baricitinib (pink).


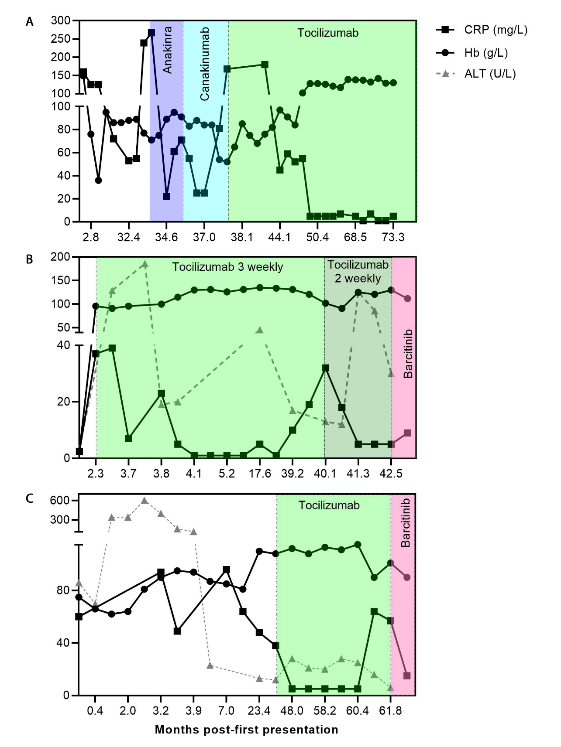


**Supplementary Figure 2**

Representative histograms demonstrating phosphorylated p65 (P-p65) phycoerythrin (PE) fluorescence intensity in lymphocytes from patient A-II-2 and healthy control after 20 minutes stimulation with: A) tumor necrosis factor; B) lipopolysaccharides; and C) interleukin-1β. Normalized count shown.

**
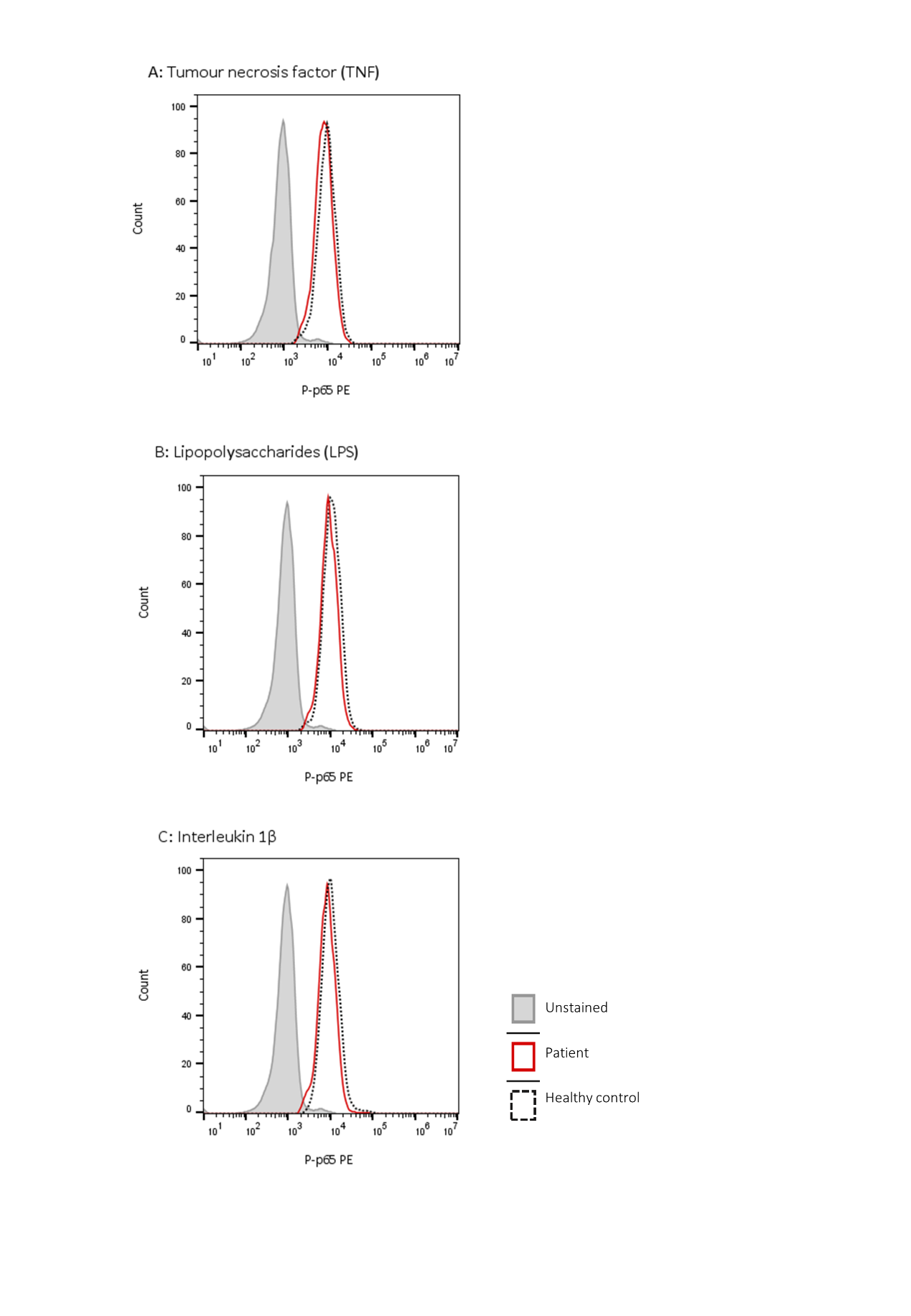
**

**Supplementary Figure 3**

**Root mean square deviation (RMSD) and principal component analysis (PCA) for IRAK4 and its D320Y mutant with four inhibitors.** The RMSD and PCA of IRAK-4 and p.D320Y mutants with four ligands (A) zimlovisertib (B) emavuseritib (C) Bay1830839 and (D) GS5718 showed that the p.D320Y mutation leads to increased scattering in conformational space and RMSD deviation. The IRAK-4 p.D320Y mutant demonstrated marked deviations with the ligands zimlovisertib, emavuseritib, and GS5718. The complex with Bay1830839, showed similar trends initially but merely deviates 125 ns onwards from IRAK-4 during the simulation. PCA analysis revealed scattering in conformational subspace of the p.D320Y IRAK-4 mutant, indicating a decrease in conformational stability in the presence of inhibitors. The dispersed conformers in the IRAK-4 p.D320Y mutant with four ligands cause the conformational subspace to be scattered due to continuous periodic jumps from one state to another. Continuous color representations from blue to yellow indicate conformational changes during the simulation.


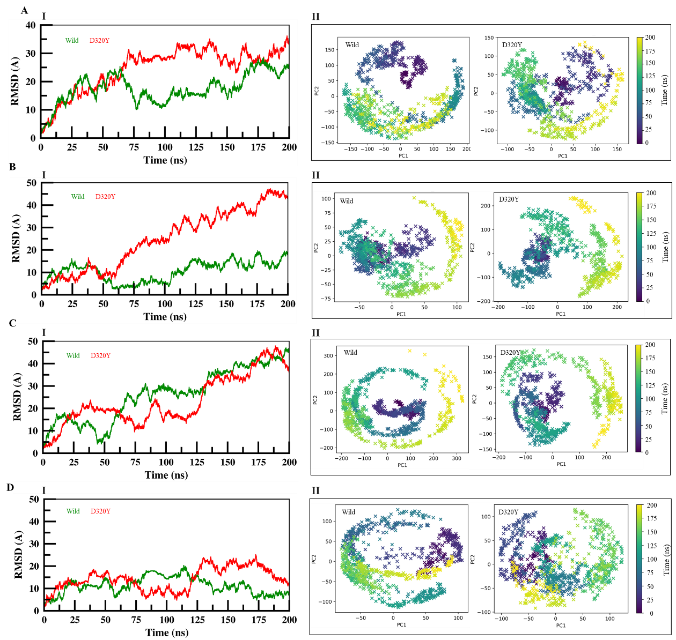


**Supplementary Figure 4.**

**Superposition of different snapshots of the protein-ligand complex extracted at a time point of 25, 75, 125 and 175 ns.** Behavior of (A) zimlovisertib, (B) emavuseritib, (C) Bay1830839 and (D) GS5718 at the binding site in the presence of the D320Y mutation compared to its wild type. Changes in the geometry of important residues are highlighted in wild type and p.D320Y mutant IRAK-4 proteins. The IRAK-4 ligand is green, while the p.D320Y ligand is shown in red.


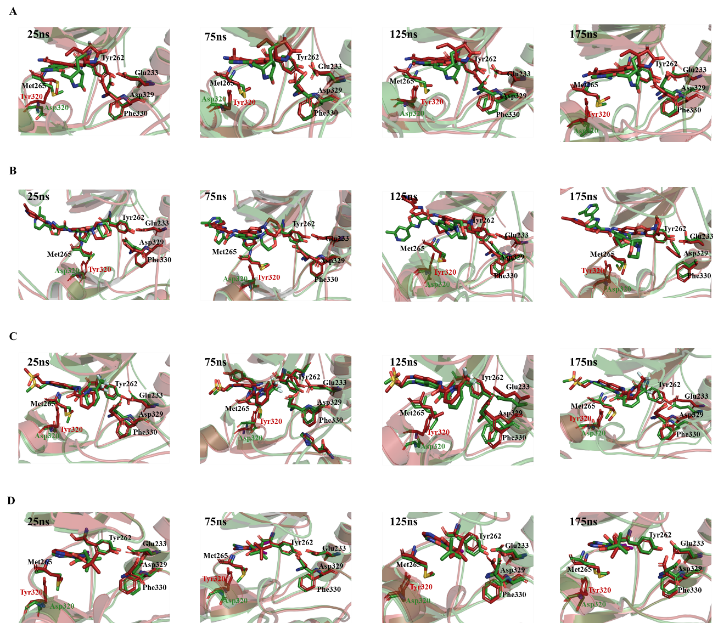


**Supplementary Figure 5.**

**End-state free energies of the ligands toward IRAK4 and its D320Y mutant.** The end-sate free energy of ligands **(A)** zimlovisertib **(B)** emavuseritib **(C)** Bay1830839 and **(D)** GS5718 toward IRAK4 and its D320Y mutant was computed. D320Y mutation decreases inhibitor binding affinity. The binding affinity of zimlovisertib, emavuseritib, Bay1830839 and GS5718 were reduced by -3, -2, -4 and -5 kcal/mol, respectively, by mutation of D320Y.


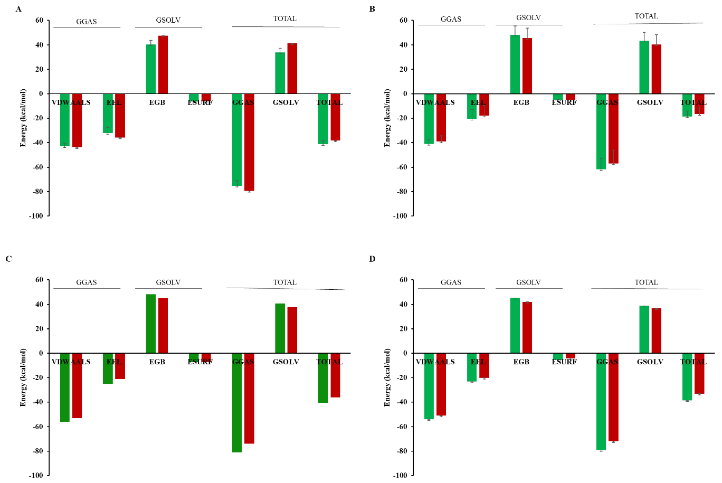


**Supplementary** **Table 1. Clinical laboratory results***

|  |  |  | **FAMILY A** | | **FAMILY B** | | |
| --- | --- | --- | --- | --- | --- | --- | --- |
|  | **Units** | **Reference range** | II-1 | II-2 | II-1 | II-2 | II-3 |
| **Haematology** |  |  |  |  | **♦Pre-/post- splenectomy** |  |  |
| Hb | g/L | 115 – 145 | **76** | **95** | **107**/**109** | **64** | **80** |
| RBC | ×10^12^/L | 3.90 – 5.30 | 3.71 | 5.16 | 3.94/2.85 | 2.85 | 4.22 |
| MCV | fL | 75.0 – 87.0 | **63** | **58.1** | **78**/**73.6** | **88.2** | **70.9** |
| PLT | ×10^9^/L | 150 – 450 | 171 | 317 | **73/1105** | 243 | 205 |
| WBC | ×10^9^/L | 5.0 – 15.0 | 7.96 | 7.59 | 7.5/14.33 | 5.5 | 10.74 |
| Neutrophils | ×10^9^/L | 1.5 – 8.5 | 3.44 | 2.98 | 3.8/5.0 | 1.3 | 2.93 |
| Lymphocytes | ×10^9^/L | 2.0 – 9.5 | 2.77 | 3.99 | 2.8/7.51 | 4.7 | 7.04 |
| Monocytes | ×10^9^/L | 0.3 – 1.5 | 0.96 | 0.81 | 0.6/**1.72** | UK | 0.67 |
| Eosinophils | ×10^9^/L | 0.3 – 0.8 | 0.06 | 0.08 | 0.0/0.04 | UK | 0.08 |
| Basophils | ×10^9^/L | 0.00-0.20 | 0.04 | 0.03 | 0.0/0.06 | UK | 0.02 |
| Reticulocytes | ×10^9^/L | 10-105 (0.8-1.5%) | 0.15% | ND | 5%/0.1% | 7% | ND |
| Blood film | N/A | N/A | UK | Microcytic, hypochromic red cells | Red cell aniosopoikilocytosis that are microcytic and hypochromic,  thrombocytosis | Red cell anisocytosis, polychromatic macrocytes, few spherocytes, occasional tear drops | ND |
| Blood marrow aspirate | N/A | N/A | Scattered histocytes, increased erythrocyte activity, no phagocytes | ND | Megakaryocytes present in normal number.  Normal trilineage hematopoiesis *post-splenectomy | Normocellular bone marrow with megaloblastic, mildly dysplastic expanded erythropoiesis | ND |
| **Clotting** |  |  |  |  | **Pre-splenectomy** |  |  |
| PT | seconds | 9.6 – 11.8 | UK | 10.1 | 10.9 | 10.2 | ND |
| INR |  | <1.1 | 1.0 | 1.0 | 1.0 | 1.0 | ND |
| APTT | seconds | 26 – 35 | 31 | 31 | 30.9 | 27.7 | ND |
| TT | seconds | 14 – 19 | UK | 13 | UK | 14 | ND |
| Fibrinogen | g/L | 14 – 40 | **5.0** | **4.4** | **4.62** | **5.5** | ND |
| **Biochemistry** |  |  |  |  | **^♦^Pre-/post- splenectomy** |  |  |
| CRP | mg/L | <20 | **125** | **49** | **217.7/85** | **60** | **41** |
| ESR | mm/hr | 0 – 10 | **135** | **116** | UK/**97** | **50** | **100** |
| SAA | mg/L | <10 | **101** | **196** | UK/**127** | **343** | **202** |
| Ferritin | μg/L | 8.6-74.0 | **363** | **88.4** | UK/**467** | 59.4 | 73.2 |
| LDH | U/L | 192-321 | **446** | **517** | 237**/702** | **354** | ND |
| Albumin | g/L | 35 - 52 | 43 | 40 | **21**/43 | 38 | 39 |
| Total bilirubin | μmol/L | <18 | 9 | 8 | 20/7 | 38 | 14 |
| ALT | U/L | 5 – 45 | **172** | **129** | <6**/58** | **86** | 43 |
| AST | U/L | 20 – 60 | 36 | 58 | UK/UK | 17 | ND |
| ALP | U/L | 150 – 380 | **99** | **121** | **104**/185 | 186 | 204 |
| Triglycerides | mmol/L | 0.36 – 1.31 | ND | **2.24** | UK/ND | **2.21** | ND |

*Tests on first presentation. ^♦^Investigations pre- (1 month prior to surgery) and post- (most recent bloods) following splenectomy. UK – Unknown; N/A – Not applicable; NI – Not indicated; ND – Not done; Hb – haemoglobin; RBC – red blood cells; MCV – mean cell volume; PLT – platelets; WBC – white blood cells; PT – prothrombin time; INR – international normalised ratio; activated partial thromboplastin time; TT-thrombin time; CRP – C-reactive protein; ESR – erythrocyte sedimentation rate; SAA – serum amyloid A; LDH – lactate dehydrogenase; ALT – alanine aminotransaminase; ASP – aspartate transaminase; ALP – alkaline phosphatase.

**Supplementary Table 2. Imaging and treatment**

|  | **FAMILY A** | | **FAMILY B** | | |  |
| --- | --- | --- | --- | --- | --- | --- |
|  | II-1 | II-2 | II-1 | II-2 | II-3 | |
| **Imaging** |  |  |  |  |  | |
| Abdominal ultrasound | Hepatosplenomegaly | Hepatosplenomegaly | Hepatosplenomegaly | Hepatosplenomegaly | Splenomegaly | |
| Chest X-ray | Normal | Perihilar bronchial wall thickening | Normal | Normal | NI | |
| CT - head | NI | Bilateral temporal subcortical calcifications. | NI | Bilateral temporal cortical calcifications. | NI | |
| MRI head | Evidence of past choroid plexus hemorrhage (probably incidental). No calcification and no evidence of past or current inflammation. | Bilateral (left>right) temporal encephalitis with vasogenic oedema and subcortical and cortical enhancement. | Normal; no evidence of past or current neuroinflammation. | Bilateral temporal cortical hyperintensity and enhancement.  Volume reduction of right temporal lobe and hippocampus. | Symmetrical signal abnormality in the medial temporal regions with signs, temporal stems and white matter adjacent to frontal horns with some enhancement. | |
| MRI Spine | NI/ND | Normal | Normal | Normal | NI/ND | |
| Echocardiography | LV hypertrophy, small pericardial effusion, LVEF 75% | Normal | Normal | Mild concentric LV hypertrophy, good biventricular systolic function. Trivial TR and PR | NI | |
| **Treatment** |  |  |  |  |  | |
|  | 1. Anakinra –   up to 6.25 mg/kg/day   1. Canakinumab –   up to 6 mg/kg every 8 weeks   1. Tocilizumab –   162 mg s-c every 3 weeks. | 1. Tocilizumab* –   162 mg s-c every 3 weeks up-titrated to every 2 weeks after 3 years   1. Baricitinib –   2 mg three time daily increased to 4 mg twice daily after 72h together with 40 mg daily prednisolone, weaned by 5 mg per week to 5 mg daily; plus prednisolone 2 mg/kg/day, unable to wean below 0.25 mg/kg/day) | Splenectomy  Penicillin V prophylaxis | 1. Tocilizumab* – 12 mg/kg every 2 weeks IV, reduced to 6 mg/kg due to episodes of febrile neutropenia; then switched to 162 mg s-c 2 weekly due to venous access issues 2. Baricitinib 2 mg thrice daily with 30 mg prednisolone daily weaning planned by 5 mg per week to 5 mg daily. Unable to wean below 15 mg daily oral prednisolone due to high CRP. | 1. Baricitinib 2 mg three times daily with 15 mg prednisolone weaning by 2.5 mg every 2 weeks to 5 mg daily. | |

N/A – Not applicable; NI – Not Indicated; ND – Not Done; CT – Computed tomography; MRI – magnetic resonance imaging;; LV – left ventricular; LVEF – left ventricular ejection fraction; TR – tricuspid regurgitation; PR – pulmonary regurgitation; s-c – subcutaneous; IV – intravenous. *Standard tocilizumab dosing IV is 12 mg/kg for weight <30 kg and 8 mg/kg for weight >30 kg, given every 2 weeks. Tocilizumab s-c dosing is 162 mg every 2 weeks for weight <30 kg and every week for weight >30 kg. Dose reduction to 4 mg/kg IV (slowly increasing to 8 mg/kg) or switching to s-c treatment is advised in the event of neutropaenia or thrombocytopaenia.

**Supplementary Table 3. Immunological investigations**

|  |  |  | **FAMILY A** | | **FAMILY B** | | |
| --- | --- | --- | --- | --- | --- | --- | --- |
|  | **Units** | **Reference range** | II-1 | II-2 | II-1  Post-splenectomy | II-2 | II-3 |
| **Autoimmune autoantibodies** |  |  |  |  |  |  |  |
| Rheumatoid factor | IU/ml | 0-15.9 | <20 | ND | ND | <9.4 | ND |
| ANA | IU/ml | 0-1.99 | Negative | ND | ND | Negative | ND |
| ANCA – PR3 | IU/ml | 0-3.49 | 1.9 | <0.2 | ND | <0.6 | ND |
| ANCA – MPO | IU/ml | 0-3.49 | 1.2 | <0.2 | ND | <0.2 | ND |
| ASOT | IU/ml |  | <12.6 | ND | ND | <12.7 | ND |
| ACE | U/L | 0-90 | 21 | ND | ND | 21 | ND |
| Anti-phospholipid antibodies |  |  | ND | ND | ND | Negative | ND |
| **Routine Immunology** |  |  |  |  |  |  |  |
| Lymphocyte subsets |  |  | Normal | Normal | Normal | Normal | ND |
| Vaccine responses:  Tetanus Ab  Pneumococcal | iu/ml | >0.1  13/13 | Normal  Normal | Normal  Normal | 0.13  Normal | 0.37  Normal | ND |
| PHA stimulation |  |  | Normal | Normal | Normal | ND | ND |
| C3 | g/L | 0.75-1.65 | 1.65 | 1.32 | ND | ND | ND |
| C4 | g/L | 0.14-0.54 | 0.30 | 0.22 | ND | ND | ND |
| **Immunoglobulins** |  |  |  |  |  |  |  |
| IgG | g/L | 4.9 – 16.1 | **26.1** | **16.7** | **20.0** | 9.42 | ND |
| IgA | g/L | 0.4 – 2.0 | 1.6 | 1.42 | **2.84** | 0.68 | ND |
| IgM | g/L | 0.5 – 2.0 | **3.3** | 2.16 | 1.66 | 1.38 | ND |
| IgD | KU/L | 2 - 100 | 26 | ND | ND | ND | ND |
| IgE | KU/L | <52 | ND | ND | ND | 11 | ND |
| **Serum cytokines (research assay)** |  |  |  |  |  |  |  |
| TNF ALPHA | pg/ml | No validated | 3.92 | 2.94 | 3.07 | 3.04 | 3.53 |
| IL-1 BETA | pg/ml | reference | 0.17 | 0.23 | 0.19 | 0.32 | 0.24 |
| IL-6 | pg/ml | range | 19.81 | 13.47 | 29.16 | 89.37 | 39.56 |
| IL-8 | pg/ml |  | 78.55 | 48.36 | 2.86 | 2.99 | 2.73 |
| MCP-1 | pg/ml |  | 373.9 | 223.52 | 205.29 | 188.45 | 223.24 |
| IP-10 | pg/ml |  | 34.47 | 40.6 | 326.65 | 144.65 | 27.94 |
| IFN-GAMMA | pg/ml |  | 5.4 | 6.14 | 4.15 | 1.59 | 8.15 |
| IFN-ALPHA-2B | fg/ml |  | 21.66 | 104.59 | 19.07 | 20.15 | 90.6 |
| IFN-BETA | fg/ml |  | 105.78 | 80.51 | 230.95 | 176.26 | 680.07 |
| **CSF Cytokines** |  |  |  |  |  |  |  |
| TNF ALPHA | pg/ml | <50 | ND | <50 | ND | <50 | ND |
| IL-2 | pg/ml | <50 | ND | <50 | ND | <50 | ND |
| IL-4 | pg/ml | <50 | ND | <50 | ND | <50 | ND |
| IL-6 | pg/ml | <50 | ND | **225** | ND | **107** | ND |
| IL-10 | pg/ml | <50 | ND | <50 | ND | <50 | ND |
| IFN GAMMA | pg/ml | <50 | ND | <50 | ND | <50 | ND |
| IFN ALPHA | IU/ml | <2 | ND | <2 | ND | <2 | ND |
| **IFN gene signature assay** |  |  | 1^st^/2^nd^ assay |  |  |  | 1^st^/2^nd^ assay |
| CXCL10 |  | 0.28 – 5.31 | 2.79/2.29 | 0.26 | 3.3 | 1.08 | 3.62/3.27 |
| CXCL9 |  | 0.29 – 2.39 | 3.35/0.76 | 0.21 | 0.75 | 0.28 | 0.28/0.37 |
| IFI27 |  | 0.09 – 2.24 | **26.0/7.51** | **6.04** | **3.1** | **4.68** | **111.13/395.87** |
| IFI44L |  | 0.24 – 7.18 | 4.09/5.08 | 0.08 | 6.27 | 1.38 | 5.67/5.64 |
| IFIT1 |  | 0.17 – 5.84 | 4.87/1.44 | 0.48 | 2.31 | 1.28 | 3.13/2.23 |
| IFN B1 |  | 0.23 – 14.12 | 52.02/0.18 | 0.35 | 0.29 | 0.12 | 0.42/0.55 |
| IFN GAMMA |  | 0.33 – 2.59 | 0.15/1.0 | 0.12 | 2.05 | 0.40 | 1.82/1.67 |
| IL-18 |  | 0.67 – 1.30 | 0.75/0.32 | 0.54 | 0.99 | 0.70 | 0.26/0.36 |
| RSAD2 |  | 0.19 – 6.86 | 2.67/0.01 | 0.15 | 2.41 | 0.75 | 3.37/1.59 |
| SIGLEC1 |  | 0.17 – 4.66 | 3.22/0.59 | 0.23 | 1.77 | 0.42 | 1.63/1.17 |
| **Other** |  |  |  |  |  |  |  |
| Metagenomics (virus/bacteria/fungi) |  |  | ND | Negative | ND | Negative | ND |
| CMV/EBV/Adenovirus/HHV6 PCR |  |  | Negative | Negative | HHV6 +ve (neonate) | HHV6 (neonate) +ve | ND |
| Soluble CD25 | pg/ml | <2500 | 2979 | ND | 2211 | ND | ND |
| ADA2 activity | U/L | 12-18 | ND | 12.68 | ND | 7.74 | ND |

*Pneumococcal & tetanus. Ig – Immunoglobulin; IFN – interferon; TNF – tumor necrosis factor; IL – interleukin; MCP – monocyte chemoattractant protein; IP - Interferon gamma inducible protein; CXCL - C-X-C motif ligand; IFI – Interferon induced; IFIT - interferon induced protein with tetratricopeptide repeats; RSAD2 - radical S-adenosyl methionine domain containing 2; SIGLEC1 - sialic acid binding Ig-like lectin 1; CMV – cytomegalovirus; EBV – Epstein Barr Virus; HHV6 human herpesvirus 6. +ve – positive.

**Supplementary Table 4. Additional neurological investigations**

|  | **Units** | **Reference range** | A-II-2 | B-II-2 |
| --- | --- | --- | --- | --- |
| **CSF analysis** |  |  |  |  |
| Appearance |  |  | Clear, colorless | Clear, colorless |
| WCC | x10^6/L | <1 | <1 | <1 |
| RCC | x10^6/L | 1 | <1 | <1 |
| Glucose | mmol/L |  | 2.9 | 2.9 |
| Protein | g/L | 0.15 - 0.45 | 0.57 | 0.16 |
| Lactate | mmol/mol |  | 1.2 | 1.2 |
| Gram stain |  |  | Negative | Negative |
| Viral PCR (VZV, HSV1&2, HHV6, HHV7, enteroviruses, parechovirus) |  |  | Negative | Negative |
| Bacterial PCR (meningococcus, pneumococcus) |  |  | Negative | Negative |
| Bacterial PCR 16s & fungal PCR 18s |  |  | Negative | Negative |
| Metagenomics |  |  | Negative | Negative |
| Oligoclonal bands |  |  | ND | Negative |
| **Encephalitis associated autoantibodies (CSF)** |  |  |  |  |
| Anti- AMPAR 1/2, GABA-B, GAD, LGI1, CASPR2, MOG, NMDAR, AQUA4 |  |  | Negative | Negative |
| **Paraneoplastic antibodies** |  |  |  |  |
| Anti-Ro, Hu, Ri |  |  | ND | Negative |
| **CSF Neurotransmitters and biochemistry** |  |  |  |  |
| 5-HIAA | nmol/L | 58-220 | 91 | 113 |
| HVA | nmol/L | 71-565 | 331 | 303 |
| Pyridoxal phosphate (Vitamin B6) | nmol/L | 10-37 | 31 | 12 |
| Dihydrobiopterin | nmol/L | 0.4-13.9 | 4.1 | 5.8 |
| Tetra hydrobiopterin | nmol/L | 9-39 | 22 | 21 |
| Total neopterin | nmol/L | 7-65 | 53 | 42 |
| **Other** |  |  |  |  |
| EEG |  |  | Background cortical rhythms are slow and higher in amplitude for the patient's age and the right cerebral hemisphere is lower in amplitude than the left. The findings are in keeping with a mild-moderate encephalopathy of non-specific etiology. | Continuous high amplitude irregular slow activity over the right hemisphere. Occasional sharp waves over the right posterior and mid-temporal region |
| **Brain Biopsy** |  |  |  | Right temporal gyrus biopsy & immunohistochemistry |
| Histology |  |  | ND | Pathological tissue with scattered inflammatory cells (CD3+ T cells in perivascular distribution, diffuse marked upregulation of microglial and macrophage activation), prominent reactive gliosis with reactive astrocytes and disrupted cortical cytoarchitecture. Foci of calcification and scattered mineralized neurons. No features of vasculitis, infective process, dysplasia or neoplasia. |
| Microscopy |  |  |  | Scanty white blood cells |
| Culture |  |  |  | No growth |
| Gram stain |  |  |  | Negative |
| Grocott/PAS/DPAS stain for fungi |  |  |  | Negative |
| AAFB/Kinyoun stain for mycobacteria |  |  | ND | Negative |
| Viral PCR (VZV, HSV1&2, HHV6, HHV7, enteroviruses, parechovirus) |  |  |  | Negative |
| Bacterial PCR 16s |  |  |  | Negative |
| Fungal PCR 18s |  |  |  | Negative |

ND – Not done; WCC – white cell count; RCC – red cell count; VZV -varicella zoster virus; HSV – herpes simplex virus; HHV – human herpesvirus; PCR – polymerase chain reaction; AMPAR - α-amino-3-hydroxy-5-methyl-4-isoxazolepropionic acid receptor; GABA-B - gamma aminobutyric acid-B, GAD - glutamate decarboxylase; LGI1 - Leucine-rich glioma-inactivated 1; CASPR2 - contactin-associated protein 2; MOG- myelin oligodendrocyte glycoprotein; NMDAR -N-methyl-D-aspartate receptor AQUA4 – aquaporin 4;, 5-HIAA -5-hydroxy indole acetic acid; HVA - homovanillic acid; EEG – electroencephalogram; PAS – periodic acid-Schiff stain; DPAS – periodic acid-Schiff stain with diastase.

**Supplementary Table 5. Protein Contacts Panel for IRAK-4 residue D320**

Protein contacts panel for wild type residue D320 (Asp320) of the IRAK4 protein showing the interacting residues, the energy of the atomic interaction (kcal/mol) and the distance (Å), within a radius of 4.5 Å. Strong interactions can be seen between the polar aspartate and nearby polar residues Thr324 and Asn267 and an lesser extent the non-polar Met265.

| **Type of Contact** | **Residue** | **Interacting residue** | **Energy** | **Distance** |
| --- | --- | --- | --- | --- |
| DH (D: distance; H: hydrogen) | Asp320 | Thr324 | -8.64 | 3.8 |
| DH (D: distance; H: hydrogen) | Asp320 | Asn267 | -3.5 | 3.6 |
| D (D: distance) | Asp320 | Met265 | -0.8 | 3.9 |
| DH (D: distance; H: hydrogen) | Asp320 | Lys326 | 0.6 | 3.9 |
| D (D: distance) | Asp320 | Pro266 | 1.3 | 4.1 |

**Supplementary Table 6. Protein Contacts Panel for IRAK-4 variant Y320**

Protein contacts panel for variant residue Y320 (Tyr320) of the IRAK4 protein showing the interacting residues, the energy of the atomic interaction (kcal/mol) and the distance (Å), within a radius of 4.5 Å. The variant tyrosine has a hydrophobic side chain the predominantly interacts with Met265 and Asn267.

| **Type of Contact** | **Residue** | **Interacting residue** | **Energy** | **Distance** |
| --- | --- | --- | --- | --- |
| DH (D: distance) | Tyr320 | Met265 | -0.7 | 4.0 |
| DH (D: distance) | Tyr320 | Pro266 | 1.07 | 4.14 |
| D (D: distance; H: hydrogen) | Tyr320 | Asn267 | -3.6 | 3.67 |
| D (D: distance) | Tyr320 | Lys290 | 2.03 | 3.7 |
| DH (D: distance; H: hydrogen) | Tyr320 | Thr324 | 399.04 | 3.6 |
| D (D: distance) | Tyr320 | Lys326 | 1.64 | 4.18 |

**References for Supplementary Methods & Results**

1. Chaudhury, S., Lyskov, S. & Gray, J.J. PyRosetta: a script-based interface for implementing molecular modeling algorithms using Rosetta. *Bioinformatics* **26**, 689-691 (2010).

2. Ahmad, B. & Choi, S. Unraveling the Tomaralimab Epitope on the Toll-like Receptor 2 via Molecular Dynamics and Deep Learning. *ACS Omega* **7**, 28226-28237 (2022).

3. Ahmad, B., Batool, M., Ain, Q.U., Kim, M.S. & Choi, S. Exploring the Binding Mechanism of PF-07321332 SARS-CoV-2 Protease Inhibitor through Molecular Dynamics and Binding Free Energy Simulations. *Int J Mol Sci* **22** (2021).

4. Huang, J. *et al.* CHARMM36m: an improved force field for folded and intrinsically disordered proteins. *Nature methods* **14**, 71-73 (2017).

5. Abraham, M.J. *et al.* GROMACS: High performance molecular simulations through multi-level parallelism from laptops to supercomputers. *SoftwareX* **1**, 19-25 (2015).

6. Leszczynski, J. & Shukla, M.K. *Practical aspects of computational chemistry*. Springer, 2012.

7. Berendsen, H.J., Postma, J.v., Van Gunsteren, W.F., DiNola, A. & Haak, J.R. Molecular dynamics with coupling to an external bath. *The Journal of chemical physics* **81**, 3684-3690 (1984).

8. Bussi, G., Donadio, D. & Parrinello, M. Canonical sampling through velocity rescaling. *The Journal of chemical physics* **126**, 014101 (2007).

9. Essmann, U. *et al.* A smooth particle mesh Ewald method. *The Journal of chemical physics* **103**, 8577-8593 (1995).

10. Páll, S. & Hess, B. A flexible algorithm for calculating pair interactions on SIMD architectures. *Computer Physics Communications* **184**, 2641-2650 (2013).

11. Hess, B., Bekker, H., Berendsen, H.J. & Fraaije, J.G. LINCS: a linear constraint solver for molecular simulations. *Journal of computational chemistry* **18**, 1463-1472 (1997).

12. DeLano, W.L. The PyMOL molecular graphics system. [*http://www*](http://www)*.pymol.org* (2002).

13. McGibbon, R.T. *et al.* MDTraj: A Modern Open Library for the Analysis of Molecular Dynamics Trajectories. *Biophys J* **109**, 1528-1532 (2015).

14. Pedregosa, F. *et al.* Scikit-learn: Machine learning in Python. *the Journal of machine Learning research* **12**, 2825-2830 (2011).

15. Valdes-Tresanco, M.S., Valdes-Tresanco, M.E., Valiente, P.A. & Moreno, E. gmx_MMPBSA: A New Tool to Perform End-State Free Energy Calculations with GROMACS. *J Chem Theory Comput* **17**, 6281-6291 (2021).

16. Ahmad, B., Batool, M., Kim, M.S. & Choi, S. Computational-Driven Epitope Verification and Affinity Maturation of TLR4-Targeting Antibodies. *Int J Mol Sci* **22** (2021).

17. Hollingsworth, S.A. & Dror, R.O. Molecular Dynamics Simulation for All. *Neuron* **99**, 1129-1143 (2018).

18. Maximova, T., Moffatt, R., Ma, B., Nussinov, R. & Shehu, A. Principles and Overview of Sampling Methods for Modeling Macromolecular Structure and Dynamics. *PLoS Comput Biol* **12**, e1004619 (2016).

19. Lee, K.L. *et al.* Discovery of Clinical Candidate 1-{[(2S,3S,4S)-3-Ethyl-4-fluoro-5-oxopyrrolidin-2-yl]methoxy}-7-methoxyisoquinoli ne-6-carboxamide (PF-06650833), a Potent, Selective Inhibitor of Interleukin-1 Receptor Associated Kinase 4 (IRAK4), by Fragment-Based Drug Design. *J Med Chem* **60**, 5521-5542 (2017).

20. Wang, L. *et al.* Conformational flexibility and inhibitor binding to unphosphorylated interleukin-1 receptor-associated kinase 4 (IRAK4). *J Biol Chem* **294**, 4511-4519 (2019).

21. Li, D. & Ji, B. Protein conformational transitions coupling with ligand interactions: simulations from molecules to medicine. *Medicine in Novel Technology and Devices* **3**, 100026 (2019).
